# Genetic load and viability of a future restored northern white rhino population

**DOI:** 10.1111/eva.13683

**Published:** 2024-04-11

**Authors:** Aryn P. Wilder, Cynthia C. Steiner, Sarah Hendricks, Benjamin C. Haller, Chang Kim, Marisa L. Korody, Oliver A. Ryder

**Affiliations:** ^1^ Conservation Genetics San Diego Zoo Wildlife Alliance Escondido California USA; ^2^ Institute for Interdisciplinary Data Sciences University of Idaho Moscow Idaho USA; ^3^ Department of Computational Biology Cornell University Ithaca New York USA; ^4^ University of California Santa Cruz Genomics Institute Santa Cruz California USA; ^5^ Department of Neurological Surgery University of California San Francisco California USA

**Keywords:** fitness, genetic load, genetic restoration, in vitro conservation, runs of homozygosity, simulation

## Abstract

As biodiversity loss outpaces recovery, conservationists are increasingly turning to novel tools for preventing extinction, including cloning and in vitro gametogenesis of biobanked cells. However, restoration of populations can be hindered by low genetic diversity and deleterious genetic load. The persistence of the northern white rhino (*Ceratotherium simum cottoni*) now depends on the cryopreserved cells of 12 individuals. These banked genomes have higher genetic diversity than southern white rhinos (*C. s. simum*), a sister subspecies that successfully recovered from a severe bottleneck, but the potential impact of genetic load is unknown. We estimated how demographic history has shaped genome‐wide genetic load in nine northern and 13 southern white rhinos. The bottleneck left southern white rhinos with more fixed and homozygous deleterious alleles and longer runs of homozygosity, whereas northern white rhinos retained more deleterious alleles masked in heterozygosity. To gauge the impact of genetic load on the fitness of a northern white rhino population restored from biobanked cells, we simulated recovery using fitness of southern white rhinos as a benchmark for a viable population. Unlike traditional restoration, cell‐derived founders can be reintroduced in subsequent generations to boost lost genetic diversity and relieve inbreeding. In simulations with repeated reintroduction of founders into a restored population, the fitness cost of genetic load remained lower than that borne by southern white rhinos. Without reintroductions, rapid growth of the restored population (>20–30% per generation) would be needed to maintain comparable fitness. Our results suggest that inbreeding depression from genetic load is not necessarily a barrier to recovery of the northern white rhino and demonstrate how restoration from biobanked cells relieves some constraints of conventional restoration from a limited founder pool. Established conservation methods that protect healthy populations will remain paramount, but emerging technologies hold promise to bolster these tools to combat the extinction crisis.

## 1 INTRODUCTION

Human activities have led to a worldwide loss of biodiversity (Cardinale et al., 2012). An estimated 22% of mammal populations are at risk of extinction (http://www.iucnredlist.org). With the alarming pace of global biodiversity loss, conservationists must increasingly turn to nontraditional and last‐ditch methods for saving species, including genetic restoration of populations from banked gametes or cryopreserved cell lines (Ben‐Nun et al., 2011; Hildebrandt et al., 2021; Korody et al., 2021). Cloning, in vitro fertilization, and eventually in vitro gametogenesis from induced pluripotent stem cells (iPSCs) can restore lost genetic diversity into a population or even an extinct species, providing a second chance for recovery (Gross, 2021; Saragusty et al., 2016). But just as the viability of a recovering population hinges on habitat availability and the mitigation of threats (Crees et al., 2016), it may also depend on the genomic health of the population (Kardos et al., 2021, 2023). If cryopreserved individuals represent the genomic diversity of pre‐collapse populations, the genomes of banked cells could harbor the same intrinsic constraints (e.g., low genetic diversity, maladaptation, inbreeding depression) that may have contributed to the demise of their original population. Thus, assessment of the genomic health of cryopreserved genomes and their capacity for restoring a viable population can help guide the use of cellular‐based conservation techniques.

The fitness and viability of small populations are influenced by the distribution, frequency, and severity of deleterious genetic load (Bertorelle et al., 2022; Kardos et al., 2021). As genetic drift exceeds the strength of purifying selection, deleterious alleles can increase in frequency and become fixed, causing a “mutational meltdown” (Dussex et al., 2023; Lynch et al., 1995). Although smaller populations tend to accumulate mildly to moderately deleterious alleles at higher frequencies, large populations harbor more highly deleterious alleles segregating at low frequencies, termed inbreeding load or masked load (Bertorelle et al., 2022; Hedrick & García‐Dorado, 2016). Masked or inbreeding load can make large populations more susceptible to inbreeding depression during severe population bottlenecks, as recessive, highly deleterious alleles are brought together in a homozygous state under inbreeding and genetic drift (Hedrick & García‐Dorado, 2016). The quantification of genome‐wide genetic load can provide a fitness‐relevant assessment of genomic health, susceptibility to inbreeding depression and the capacity for recovery of endangered species (Robinson et al., 2022; Wilder et al., 2023).

**FIGURE 1:**
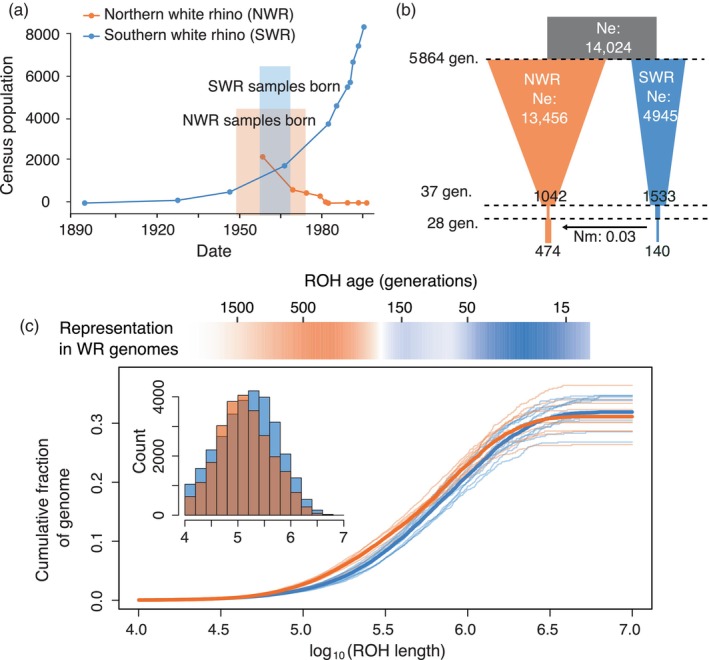
(a) Census population sizes of NWR and SWR over the past two centuries. When our study individuals were born, the SWR population was recovering from <200 individuals and the NWR population was still declining. (b) The demographic model that best fit the two‐dimensional site frequency spectrum (2dSFS), showing population decline until 37 generations ago, after which both populations underwent more rapid size changes, with some evidence of very low levels of gene flow from SWR into NWR. (c) Cumulative fraction of genomes represented by ROH lengths (thin lines are individuals, thick lines are population means; inset shows raw counts across all 22 genomes). The relative abundance of ROH lengths is shown in the heatmap above, with more orange indicating higher abundance in NWR genomes and more blue indicating higher abundance in SWR. NWR genomes had smaller ROH likely stemming from older inbreeding (>500 generations ago), whereas SWR had larger ROH from recent inbreeding (as recently as ~15 generations ago).

The white rhinoceros (*Ceratotherium simum*) consists of two subspecies with very different levels of extinction risk (Emslie, 2020; Emslie & Knight, 2014). The southern white rhinoceros (SWR; *C. s. simum*), distributed across South Africa, is currently the most abundant rhinoceros in the world. Listed as Near Threatened by the International Union for the Conservation of Nature (IUCN), it is one of only two rhinos (along with the greater one‐horned rhinoceros, *Rhinoceros unicornis*) not considered Critically Endangered. Once on the brink of extinction, swift conservation efforts enabled recovery of SWR from an estimated 50–200 individuals in the early 20th century (Figure 1a; Emslie, 2020; Rookmaaker, 2000). At its recent peak in 2014, the census population reached ~20,000 individuals, but a surge in poaching beginning a decade ago reduced the population to ~18,000 (Emslie, 2020; Emslie & Knight, 2014). The northern white rhinoceros (NWR; *C. s. cottoni*), once distributed over parts of Uganda, Chad, Sudan, the Central African Republic, and the Democratic Republic of Congo, has been driven effectively to extinction by poaching (Figure 1a), with the only remaining live individuals being two related, non‐reproductive females.

The two white rhino subspecies have distinct recent population trajectories with a shared evolutionary history. Previous genomic analyses have suggested that the subspecies began diverging ~400–600 KYA, with intermittent periods of gene flow that ceased within the last 100 KYA (Moodley et al., 2020). After divergence, both subspecies had effective population sizes (*N*_*e*_) in the thousands (Moodley et al., 2020; Tunstall et al., 2018). In SWR, the recent bottleneck was followed by a recovery to roughly half the pre‐bottleneck *N*_*e*_. NWR underwent a more recent bottleneck and has not recovered. Genomes from SWR individuals sampled post‐bottleneck and recovery (born mostly mid‐20th century; Figure 1a) have lower genetic diversity than NWR individuals born early in the NWR bottleneck (Tunstall et al., 2018). Both subspecies show evidence of genomic erosion: contemporary genomes had lower genetic diversity and higher inbreeding coefficients than those sampled in the late 19th century, before the most severe population declines (Sánchez‐Barreiro et al., 2021). Yet genomic erosion has not limited recovery of the SWR population, which has grown more than 100‐fold in the past 13 decades. NWR, by contrast, faces imminent extinction.

Cryopreserved cell lines from 12 individuals, eight of which are unrelated, offer hope for reviving a viable NWR population. NWR is the focus of research programs aimed at generating viable embryos using iPSC‐derived gametes, assisted reproductive technologies (ART), and somatic cell nuclear transfer (SCNT) (Hayashi et al., 2022; Hildebrandt et al., 2021; Korody et al., 2021; Saragusty et al., 2016). Using ART with intracytoplasmic sperm injection of cryopreserved semen into oocytes, NWR embryos have already been created and cryopreserved for future transfer into a surrogate mother (Hildebrandt et al., 2018, 2021, 2023), demonstrating the feasibility of implementing these approaches in the very near future to begin restoring the NWR population.

The extent to which a future NWR population will be constrained by the limited number of potential founders (12 cell lines) is unknown. Small populations are susceptible to extinction from stochastic environmental events and Allee effects, a risk compounded by low fitness from inbreeding depression and loss of standing genetic variation (Gilpin, 1986). The genomes of NWR cell lines retain higher genetic diversity than individuals from the recovered SWR population (Tunstall et al., 2018), suggesting the future NWR founders have adequate adaptive potential. However, historically large populations with high genetic diversity may also harbor high masked load, where the fitness effects of deleterious alleles are masked in heterozygosity (Hedrick & García‐Dorado, 2016). Inbreeding is expected to convert masked genetic load into homozygous load (Bertorelle et al., 2022), reducing fitness and potentially hindering recovery. In a population established by few founders, some level of inbreeding will likely be unavoidable. However, embryos produced via cloning or in vitro gametogenesis from iPSCs can be repeatedly reintroduced to the population over multiple generations. Unlike a single founding event, the reintroduction of founders could provide a source of genetic rescue to curb inbreeding and realized load (Wisely et al., 2015).

We conducted a comprehensive examination of genetic load to gauge the potential for future inbreeding depression and low fitness that might hinder recovery of a population founded by these cell lines. We first characterized the distributions of genome‐wide deleterious variation and their relationships with the demographic histories of the two subspecies. We hypothesized the prolonged bottleneck in SWR led to an accumulation of mildly and moderately deleterious alleles and elevated homozygous genetic load, whereas the shorter bottleneck in NWR allowed the population to retain more highly deleterious variants masked in heterozygosity (Hedrick & García‐Dorado, 2016). If confirmed, this high inbreeding load may reduce fitness as a restored NWR population becomes increasingly inbred and exposed to drift. Using SWR genomes as a benchmark for viable recovery potential, we simulated genetic load and its fitness costs in NWR under two scenarios: one with a single founding event and the other with repeated reintroductions of founder genomes in subsequent generations. The comparison of independent populations with similar life‐history characteristics, shared evolutionary history, and distinct recent demographic trajectories provides a unique framework for isolating the impact of past and future population fluctuations on genetic load, and a means for assessing the potential for genetic restoration and recovery of NWR through in vitro conservation technology.

## 2 MATERIALS AND METHODS

We focused our analysis on publicly available genomes from contemporary samples (Sánchez‐Barreiro et al., 2021; Tunstall et al., 2018) to provide a detailed and in‐depth analysis of genetic load and demographic history. We excluded the historical samples of Sánchez‐Barreiro et al. (2021) to avoid limitations from low read depth and DNA damage inherent to these samples. All necessary permits from Convention on International Trade in Endangered Species (CITES), United States Fish and Wildlife Service (USFWS), United States Department of Agriculture (USDA), domestic and foreign customs, and local permits were acquired for legal possession and use of samples in this project.

### 2.1 Read mapping and SNP calling

We analyzed whole genomes of nine NWR and 13 SWR individuals (Table S1). Eight of the nine NWR individuals are represented by fibroblast cell lines in San Diego Zoo Wildlife Alliance's Frozen Zoo and six of these have been successfully reprogrammed into iPSCs (Korody et al., 2021). We downloaded whole‐genome sequence reads from these nine NWR born between 1952 and 1972, and four SWR born between 1958 and 1963, generated by Tunstall et al. (2018). Nine additional SWR samples were generated by Sánchez‐Barreiro et al. (2021), two from wild individuals born 1968–1969, six captive individuals born 2008–2011, and one wild individual born in 2012.

TruSeq3 adapters were trimmed from paired‐end reads using Trimmomatic v.0.3.2 (Bolger et al., 2014) under the following settings: seed mismatches = 2, palindrome clip threshold = 30, simple clip threshold = 10, minimum adapter length = 4, and keeping both reads. Trimmed reads were mapped to the SWR reference genome CerSimSim1.0 using bowtie2 under the very sensitive parameter settings (Langmead & Salzberg, 2012), with a maximum fragment length of 2 KB. Duplicate reads were removed using MarkDuplicates in Picard (Van der Auwera & O'Connor, 2020). Sequence read depth was higher in the more recently generated samples from Sánchez‐Barreiro et al. (2021; 21.65×, range: 18.46–25.07×) than those from Tunstall et al. (2018; 14.32×, range: 11.01–15.54×; Table S1). Genotype calls from these moderate depths of coverage are expected to have high overall concordance (>98%) with true genotype calls, but are known to underestimate heterozygous genotypes from lower coverage samples (Kishikawa et al., 2019). To minimize biases in heterozygous call rates stemming from the different read depths, we randomly subset genome‐wide reads from the higher coverage samples to 14.3× coverage (the average read depth across the Tunstall et al. (2018) samples) using Samtools (Danecek et al., 2021).

Variants were called in GATK v3.8 (Van der Auwera & O'Connor, 2020), first in all samples individually using HaplotypeCaller in gVCF mode, then across all samples combined using GenotypeGVCFs. We examined the distributions of genome‐wide SNPs and indels to select thresholds for filtering variants and retained variants that passed the following criteria, for SNPs: QD > 5, FS < 32, SOR < 3, MQRankSum > −2, MQ > 28, ReadPosRankSum > −2, and for indels: QD > 5, FS < 32, SOR < 3, ReadPosRankSum > −2. After applying filters, we retained 7,653,428 variants.

### 2.2 Polarizing ancestral and derived alleles

The ancestral and derived alleles were polarized at each variant using genomes from two black rhino subspecies, the eastern black rhino (*Diceros bicornis michaelis*) and southern black rhino (*D. bicornis minor*). Reads used for the assembly of the eastern and southern black rhino reference genomes (SRR11430189 and SRR12010331, respectively) were downloaded and mapped to the SWR reference genome using the same methods described above. We then separately called genotypes in the two black rhino genomes at all positions that were polymorphic in white rhinos. We used GATK HaplotypeCaller to generate single sample gVCFs, followed by GenotypeGVCFs. We included sites that were non‐variant in the black rhino genomes as well as variant ones. Raw variants were then filtered using the same methods as for white rhinos, by plotting the distributions of genome‐wide SNPs and indels to select hard filter thresholds. For both black rhino genomes we retained genotypes that met the following criteria: QD > 22, FS < 32, MQ > 32, MQRankSum > −5, ReadPosRankSum > −2, SOR < 3. We defined the ancestral allele as the allele that was homozygous in both black rhino genomes, ignoring sites with more than one allele in the two black rhino genomes (i.e., heterozygous sites or sites with different alleles in the two black rhino genomes). We were able to polarize 6,267,082 variants with ancestral and derived alleles.

### 2.3 Estimating heterozygosity and runs of homozygosity

We estimated runs of homozygosity (ROH) in white rhino genomes from the filtered variant set using the ROH function in bcftools v.1.8, which applies a hidden Markov model to biallelic sites to identify genomic stretches of the genome that show identical alleles on both chromosomes (Narasimhan et al., 2016). To estimate inbreeding coefficients (*F*_ROH_), we summed the lengths of ROH >1 MB (with bcftools ROH quality scores >30) across the genome and divided by the genome length. We estimated heterozygosity in PLINK v1.9 (‐‐het function) from a subset of high‐quality variants, filtering out sites with >30% missing data after removing genotypes with GQ < 20 to minimize biases stemming from differences in sequencing batches (Purcell et al., 2007). We then divided the number of heterozygous sites per sample by the gapless length of the reference genome, adjusted for the proportion of called genotypes per sample.

### 2.4 Demographic modeling

Previous analyses of the demographic history of white rhinos either fit models to a folded (i.e., non‐polarized) two‐dimensional site frequency spectrum (2dSFS; Tunstall et al., 2018), or used coalescence‐based analysis of single genomes (Moodley et al., 2020). We built on these previous analyses, testing the fit of more complex demographic models to an unfolded 2dSFS using polarized ancestral and derived alleles. We tested demographic models with the GADMA program, which uses a genetic algorithm in an unsupervised, heuristic global search of demographic models (Noskova et al., 2020). GADMA implements the moments program (Jouganous et al., 2017), which simulates the 2dSFS under different demographic scenarios and uses an optimization process to fit parameters to the data. We tested models with four time periods, where an ancestral population splits into two populations at time T1, followed by three time periods during which population sizes, growth dynamics (including instantaneous, exponential, and linear growth), and migration rates can change. The 2dSFS was generated from derived allele counts for each subspecies from the final set of SNPs with the script vcf2sfs.py (https://github.com/marqueda/SFS‐scripts). To allow for model comparison, we block bootstrapped the data over 4 MB blocks using the vcf2sfs.py script, generating 100 bootstrapped 2dSFS. The SFS was projected to 16 NWR alleles and 24 SWR alleles to account for missing genotypes. We performed five independent runs of GADMA on block bootstrapped 2dSFS under default settings and selected the model that minimized the composite likelihood Akaike's information criterion (CLAIC) across all runs. We then ran local searches on block bootstrapped data to generate confidence intervals for parameters of the best model in the GADMA program. We rescaled the parameter estimates to demographic units assuming a mutation rate of 2.1 × 10^−8^ (Moodley et al., 2020).

To infer the timing of inbreeding events that generated ROH in the genomes of the two subspecies, we examined the distributions of ROH sizes. To estimate the relative representation of ROH lengths in each subspecies, we plotted the cumulative sum of lengths of ROH in each genome and averaged the curves across individuals of each subspecies (Figure 1c). We then estimated the mean slopes of the curves within ROH intervals, using 1 MB sliding windows with 10 KB step size, comparing the slopes of the two subspecies (van der Valk et al., 2019). A steeper slope in one subspecies indicates greater representation of the given ROH length within that subspecies. Assuming 50 MB identity‐by‐descent tracts are inherited per generation, we can infer the time to a common ancestor of a tract of a given length (Thompson, 2013); thus, to estimate the number of generations since the inbreeding event that results in ROH of a given size, we divided 50 MB by the ROH length.

### 2.5 Genome annotation

We used the Comparative Annotation Toolkit (CAT; Fiddes et al., 2018) to annotate the rhinoceros alignment from progressive Cactus (Armstrong et al., 2020), using the NCBI horse (GCF_002863925.1) RefSeq annotation release 103 as reference. For input into the Augustus step in CAT, RNAseq data were generated from several archived tissues, including brain of a NWR female, testis of a NWR male, reproductive tissues of a SWR female, and fibroblast cell cultures from a NWR male. Libraries were prepared using the Illumina TruSeq stranded mRNA library prep kit and sequenced using paired‐end 76 bp reads on an Illumina Miseq. Reads that mapped to the genome (29.9 M reads totaling 2.3 Gb) using TopHat2 (Kim et al., 2013) were passed directly to the CAT pipeline. All steps used for the final consensus finding and transcript classification in the CAT pipeline were run except the Augustus PB step, and the default parameters were used in Augustus. We used BUSCO v4.0.2 to evaluate the annotation coverage (Figures S1–S6). Overall, the reference annotation had high coverage of protein coding genes (Figure S1; Laurasiatheria complete BUSCO: 98.1%), with good assembly contiguity in genic regions (Figures S2, S3) and support of introns, exons, and splice site boundaries (Figures S4–S6). Because the indel error rate was high in the annotation, a large number of genes failed to map using transMap and were instead included as AugustusCGP transcripts. We omitted all AugustusCGP gene predictions with 0–2 introns in the final annotation.

### 2.6 Estimating variant impacts and genetic load

To estimate genetic load, we used two approaches, inferring variant impacts based on (1) their effect on encoded proteins of protein coding genes, and (2) evolutionary conservation across mammalian taxa, with the assumption that mutations at sites that are more conserved across taxa are more likely to be deleterious. We examined 6.26 M variants that had ancestral/derived polarization of alleles and genotype calls in all 22 samples. We used default settings in the program snpEff (Cingolani, 2022) to predict the effects of variants in protein coding genes with our updated gene annotation. We estimated homozygous and heterozygous deleterious variants across all genes that are well conserved in mammals (i.e., genes in the BUSCO mammalia_odb10 set). To estimate evolutionary conservation, we downloaded the GERP++ track scores generated from a 34‐mammal alignment (http://mendel.stanford.edu/SidowLab/downloads/gerp/index.html; which does not include the white rhino genome). In this dataset, constraint intensity at each alignment position is quantified by a rejected substitutions (RS) score, which is the number of substitutions expected under neutrality minus the number of substitutions observed at the position. We defined evolutionarily conserved sites as those with RS >4. We used the liftOver function in the R package rtracklayer to lift over coordinates of variant sites from the white rhino genome to the human genome (hg19), then from hg19 to the earlier assembly of the human genome (hg18) used in the 34 mammalian species alignment to assign RS scores to each variant position in the white rhino genome. To add a second metric of evolutionary conservation, we also downloaded phyloP scores from the UCSC Genome Browser computed from the 34 mammalian species alignment and similarly used liftover to convert coordinates of variable sites in the white rhino genome to the hg19 reference. Results from GERP++ and phyloP were highly correlated, and thus we report only results from GERP++. We defined deleterious variants as those at evolutionarily conserved sites (RS >4), and mutations at evolutionarily conserved sites predicted to have moderate impact on protein coding genes (e.g., missense mutations) by snpEff. We considered homozygous deleterious variants to be “homozygous load” and heterozygous deleterious variants to be “heterozygous load.”

Because long ROH from recent inbreeding may harbor high levels of deleterious variants (Szpiech et al., 2013), we examined putatively deleterious alleles in ROH. Within length bins (0.1 MB ≤ ROH < 0.5 MB, 0.5 MB ≤ ROH < 1 MB, 1 MB ≤ ROH < 3 MB, and ROH ≥ 3 MB), we quantified the proportion of homozygous mutations at conserved sites within ROH, and the ratio of moderate‐impact (missense) homozygous alleles at conserved (RS > 4) sites relative to low‐impact homozygous alleles. We tested the relationship between ROH length and the proportion of deleterious alleles using linear regressions. We compared the proportion of homozygous sites with deleterious alleles within ROH relative to the proportion of homozygous sites with deleterious alleles in autozygous regions using paired *t*‐tests. We also compared the proportion of homozygous sites with deleterious alleles within ROH relative to the proportion of heterozygous sites with deleterious alleles in autozygous regions using paired *t*‐tests.

### 2.7 Functional enrichment tests

We annotated the predicted coding sequences (CDS) from gene annotations in the white rhino genome with gene ontology (GO) terms from several public databases. First, we used TransDecoder v5.5.0 (https://github.com/TransDecoder/TransDecoder) to predict protein‐coding regions in the white rhino transcripts based on the longest ORF. We searched for homologous sequences for both transcripts and predicted proteins in the SwissProt database (ftp://ftp.ebi.ac.uk/pub/databases/uniprot/) using BLASTX and BLASTP (NCBI‐BLAST‐2.9.0+), respectively (Altschul et al., 1990, 1997), retaining only top hits with an e‐value ≤1 × 10^−5^. We identified protein domains in white rhino transcripts by comparing to the Pfam‐A database (ftp://ftp.ebi.ac.uk/pub/databases/Pfam/) using HMMER‐3.2.1 (Finn et al., 2011). And finally, using homologous proteins in TRINOTATE‐v3 (Bryant et al., 2017), we combined functional annotations from the following databases: (1) GO (Ashburner et al., 2000), (2) EggNOG (Powell et al., 2012) and (3) KEGG (Kanehisa et al., 2012). TransDecoder predicted 13,419 protein‐coding genes within the 196,815 CDS transcripts. 12,658 protein‐coding genes and 154,102 transcripts mapped to proteins in the SwissProt database, and 7508 were present in the Pfam‐A database. Using the combined results of homology searches via SwissProt and Pfam, we assigned 22,749 unique GO terms across 17,662 transcripts.

We tested for enrichment of Biological Processes GO terms among genes with deleterious variation in the program ErmineJ v3.1.1 (Gillis et al., 2010). We used an over‐representation analysis (ORA) to test for GO enrichment among genes with at least one deleterious mutation relative to GO terms from all genes. We also used the gene score resampling (GSR) method, where each gene was scored by the number of deleterious variants divided by the length of coding sequences for the gene, and GO terms are ranked by the scores of genes within the associated gene set. We restricted the analysis to terms with 5–100 genes and used a mean class scoring method with 10,000 replicates.

### 2.8 Forward genomic simulations

To explore how future inbreeding might impact a restored NWR population, we simulated genetic load and its fitness cost in a population founded by banked NWR cell lines using forward genomic simulations in the program SLiM v4.0.1 (Haller & Messer, 2023). We modeled neutral and deleterious mutations under different population growth and restoration scenarios, using SWR as a reference for a viable level of realized genetic load. We modeled only neutral and deleterious mutations to avoid assumptions about the distribution of positive fitness effects and the absolute fitness of individuals. Deleterious mutations at evolutionarily conserved sites are likely to impact vital organismal functions in all environments (Christmas et al., 2023), whereas beneficial mutations may be more context and environment‐dependent, and their impacts are more likely to differ between taxa or in populations under human care. We started the simulations from eight NWR genomes with cryopreserved cell lines that can be used for genetic rescue. These individuals represent eight of the 12 individuals with cryopreserved cell lines. The four individuals not represented in our sequenced genomes were F1 or F2 offspring of three F0 individuals included in our dataset and thus would contribute little new diversity to the restored population. One sequenced genome in our dataset does not have cryopreserved cell lines and was excluded from the simulations.

We simulated two different types of genetic rescue strategies, one in which a restored population is founded by the eight cryopreserved cell lines in generation 0, and a second in which the restored population is supplemented with one randomly sampled cell line per generation. For both strategies, we simulated 10 non‐overlapping generations of non‐Wright–Fisher evolution under six population growth scenarios: 0%, 10%, 20%, 30%, 40%, and 50% growth per generation. We ran 50 replicates per scenario. Because we simulated only deleterious genetic variation across the genome, fitness values represented the cost of realized load in the genomes of individuals rather than their absolute fitness. The probability that an individual successfully reproduced was proportional to its fitness relative to others in the population. Specifically, each generation of parents was sampled with replacement weighted by their fitness, such that an individual with fitness 10% above the mean would have 10% higher than average probability of producing offspring. Population size and growth were predefined parameters rather than being emergent properties of the absolute fitness of individuals in the simulated population.

The simulations began in generation 0 with empirical data on deleterious variation in the genomes of the eight cryopreserved cell lines. We simulated all scaffolds in the genome >1 MB in length and assumed a per‐base recombination rate of 1 × 10^−8^ (Dumont & Payseur, 2008) and independent assortment of scaffolds. We reconstructed founder haplotypes from the observed genotypes of each individual at all mildly to severely deleterious variants (RS ≥ 2), plus 10,000 randomly selected neutral variants (RS < 2). For heterozygous variants, the two alleles were randomly assigned to the two haplotypes. Variants were binned into four groups reflecting their likely severity: “neutral” (−2 < RS < 2), “mild” (2 ≤ RS < 4), “moderate” (4 ≤ RS < 5.8), and “severe” (RS ≥ 5.8) and assigned selection and dominance coefficients to each bin based on estimates from the human genome (Henn et al., 2016); selection coefficients: *s*_neutral_ = 0, *s*_mild_ = −0.0001, *s*_moderate_ = −0.001, and *s*_*s*evere_ = −0.002; dominance coefficients: *h*_neutral_ = 0.5, *h*_mild_ = 0.293, *h*_moderate_ = 0.062, and *h*_severe_ = 0.033. To assess the sensitivity of the simulations to the assigned selection coefficients, we also tested the simulations with the following selection coefficients: *s*_neutral_ = 0, *s*_mild_ = −0.024, *s*_moderate_ = −0.032, and *s*_severe_ = −0.072 (Peischl et al., 2018), but found that the two sets of selection coefficients produced broadly consistent results (Figure S7) and thus we report results only from the former set. Because we were mainly interested in the short‐term fitness consequences of standing variation and not the long‐term accumulation of genetic load, we set the mutation rate to 0. Simulated genomes of the entire population were output each generation. Fitness cost was estimated as the product of the fitness effects of all deleterious variants in the genomes, (1 + s) for homozygous variants, and (1 + *h* × *s*) for heterozygous variants:

$$\prod_{i=1}^{L\left(\mathrm{hom}\right)}\left(1+s_{i}\right)\prod_{j=1}^{L\left(\mathrm{het}\right)}\left(1+h_{j}s_{j}\right)$$

Realized load was estimated by summing the selection coefficients of all homozygous derived mutations and summing the products of the dominance and selection coefficients across all heterozygous loci:

$$\sum_{i=1}^{L\left(\mathrm{hom}\right)}s_{i}+\sum_{j=1}^{L\left(\mathrm{het}\right)}h_{j}s_{j}$$

We estimated fitness and realized load of the 13 sequenced SWR genomes in the same way, using SWR as a benchmark for fitness in a related population that has successfully recovered from an extreme bottleneck.

One notable difference between our study and others is that our realized load estimates for the NWR and SWR genomes are many times higher than other studies because we included all putatively deleterious variants across the entire genome rather than restricting the analysis to the exome (e.g., Henn et al., 2016) or to ultraconserved regions (Speak et al., 2023). Estimating realized load from only the exome produces values comparable to those seen in studies of the exome or ultraconserved elements (mean realized load = 0.75 for NWR and 0.87 for SWR). Because fitness of individuals was modeled relative to the mean of the population, the absolute fitness values did not affect the outcomes of the models.

## 3 RESULTS

### 3.1 Demographic history and genomic variation

The model that best fit the observed 2dSFS for the two subspecies (Figure S8) was a scenario in which two populations split from an ancestral population with two recent periods of size changes in both populations (Figure 1b; Table S2). Under this model, an ancestral population of size *N*_anc_ = 14,024 split into two isolated populations of size NWR1 = 13,456 and SWR1 = 4945 at time T1 = 5864 generations ago. Population sizes decreased linearly until 37 generations ago to NWR1.1 = 1042 and SWR1.1 = 1533, after which both population sizes decreased instantaneously to NWR2 = 192 and SWR2 = 349. Over the next 28 generations, populations remained small (NWR3 = 474 and SWR3 = 140) with a very low level of asymmetrical gene flow (*N*_m_ = 0.031) from SWR into NWR. Assuming an eight‐year generation time (Tunstall et al., 2018), the populations began diverging ~47,000 years ago, with more recent size changes beginning 296 years ago. Using 25.5 years per generation (Moodley et al., 2020), the populations began diverging ~150,000 years ago, with more recent size changes beginning 944 years ago.

Pairwise nucleotide diversity was greater in the NWR population (pi = 1.12 × 10^−3^) than SWR (pi = 7.17 × 10^−4^), and observed heterozygosity was higher in all NWR individuals (mean = 1.06 × 10–3) compared to SWR individuals (mean = 7.41 × 10–4; *p* = 2.27 × 10^−13^; Figure S9), consistent with the larger historical *N*_e_ and with the findings of previous studies (Sánchez‐Barreiro et al., 2021; Tunstall et al., 2018). The NWR population had more private alleles than the SWR population, both by count (2,791,323 in NWR versus 1,866,849 in SWR) and as a proportion of polymorphic sites (0.63 in NWR versus 0.54 in SWR). The difference may be explained in part by asymmetrical gene flow favoring migration of alleles from SWR into NWR (Figure 1b), but a longer population bottleneck likely also led to the loss of low‐frequency, derived alleles from the SWR population.

There were more long ROH and F_ROH>1MB_ was higher in SWR (Figure 1c), reflecting inbreeding in the more recent past in SWR compared to NWR. To infer the timing of inbreeding events that contribute to ROH, we examined the distributions of ROH sizes in the two subspecies (van der Valk et al., 2019). SWR had greater representation of longer ROH (~2–3 MB). Assuming 50 MB identity‐by‐descent tracts are inherited per generation (Thompson, 2013), the more abundant 2–3 MB ROH in the SWR genome stemmed from inbreeding ~15 generations ago. By contrast, NWR had a greater representation of shorter ROH of ~100–200 KB. ROH of this size likely arose >190 generations ago (Figure 1c), potentially resulting from climate‐associated population declines during the Younger Dryas (Moodley et al., 2018). Given variation in recombination rates across the genome and across species, there is likely much variation around these estimates, but the results generally suggest that ROH in the NWR genome reflect historical demographic events in the more distant past, whereas large ROH in the SWR genome are much younger, most likely arising during the recent bottleneck.

### 3.2 Deleterious genetic load

**FIGURE 2:**
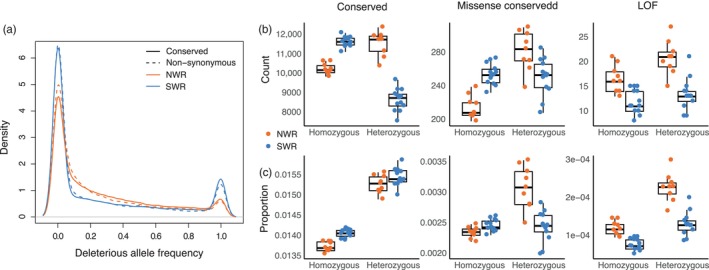
(a) Deleterious alleles tend to segregate at lower frequencies in both populations, but proportionally more conserved (RS > 4) and non‐synonymous (missense and loss of function) deleterious alleles are fixed in SWR (*p* = 0.025) than NWR. (b) Compared to NWR, SWR genomes have more homozygous and fewer heterozygous deleterious alleles at conserved and missense conserved sites, but fewer homozygous and heterozygous loss of function (LOF) alleles by count. (c) SWR genomes have proportionally more homozygous deleterious alleles at conserved and missense conserved sites than SWR, but proportionally fewer homozygous and heterozygous LOF alleles than NWR.

Two approaches to estimate genetic load produced largely consistent results, pointing to higher homozygous and fixed load in SWR and higher heterozygous and segregating load in NWR. We first estimated load based on changes at evolutionarily conserved sites in the genome (RS > 4) assumed to have moderately to severely deleterious impacts on fitness. As expected for alleles under purifying selection, moderately to severely deleterious alleles (RS > 4) tended to segregate at lower frequency in both populations. In SWR, more deleterious alleles were fixed, both in total and as a proportion of all fixed derived alleles, compared to NWR (*χ* ^2^ = 4.99, *p* = 0.026), suggesting stronger drift over the long term in SWR than in NWR (Figure 2a). By count, there were more deleterious variants segregating in the NWR population (40,034 sites with at least one deleterious allele in NWR compared to 31,821 in SWR). In individual NWR genomes, there were more deleterious alleles than in SWR genomes (*p* = 0.034), but more of these alleles were in a heterozygous state (Figure 2b). In SWR genomes, a larger proportion of homozygous sites had deleterious alleles compared to NWR (Figure 2c; *p* = 2.82 × 10^−10^), and sites with homozygous derived alleles had higher RS scores on average in SWR compared to NWR (*p* = 1.63 × 10^−5^). The proportion of heterozygous deleterious alleles was slightly but not significantly higher in SWR compared to NWR (Figure 2c; *p* = 0.051).

The results were largely similar when we examined variation by its predicted impact in protein coding genes. Here we defined deleterious alleles as derived alleles with moderate‐impact (e.g., missense) or high‐impact/loss of function (e.g., premature stop codons, frameshift mutations) effects on the protein that were also evolutionarily conserved (RS > 4). There were more moderate‐impact, evolutionarily conserved variants segregating in the NWR population than SWR (1083 vs. 931 variants, respectively). There were also more loss of function (LOF) variants segregating in the NWR population than SWR (114 vs. 74 variants, respectively). Individual SWR genomes carried more moderate‐impact homozygous alleles by count (*p* < 1 × 10^−6^), and in proportion to homozygous derived alleles in coding sequences (*p* < 1 × 10^−6^; Figure 2b). High‐impact, loss of function mutations showed the opposite pattern (Figure 2b). SWR genomes had fewer homozygous and heterozygous LOF alleles by count (*p* = 4.71 × 10^−4^ and *p* = 4.55 × 10^−5^ for homozygous and heterozygous, respectively) and in proportion to all coding variants (*p* = 4.71 × 10^−4^ and *p* = 4.55 × 10^−5^ for homozygous and heterozygous, respectively) compared to NWR, suggesting that some high‐impact alleles may have been lost from the SWR population through drift or purging. Overall, the genetic load analyses suggest that SWR genomes harbor more homozygous, moderate‐impact genetic load, whereas NWR genomes have lower realized load but higher masked load.

**FIGURE 3:**
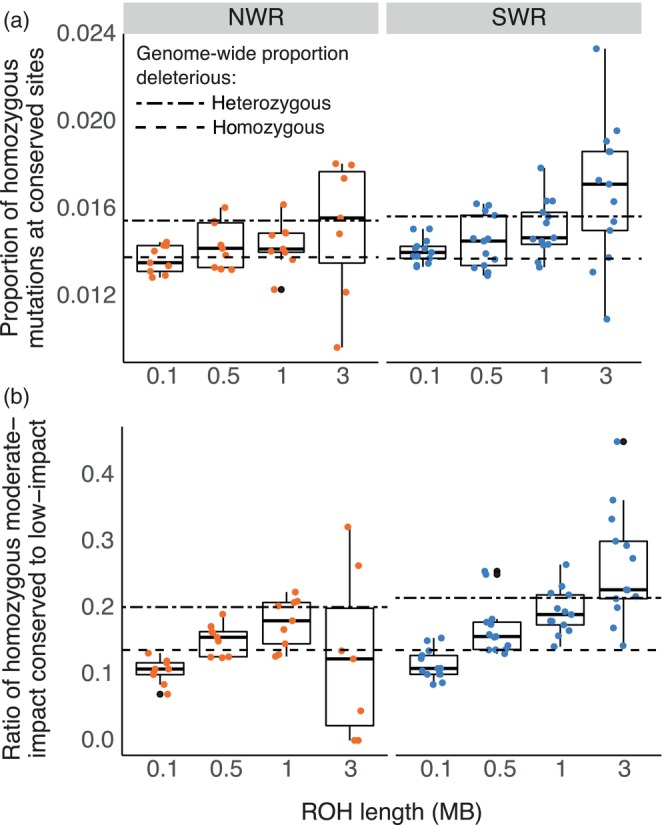
Longer ROH are enriched for homozygous deleterious mutations. The proportion of homozygous mutations at conserved sites (a) and the ratio of homozygous moderate‐impact conserved (RS > 4) to homozygous low‐impact sites (b) significantly increased with ROH length (bins: 0.1 MB ≤ ROH < 0.5 MB, 0.5 MB ≤ ROH < 1 MB, 1 MB ≤ ROH < 3 MB, and ROH ≥ 3 MB) in both white rhino subspecies. There were likely too few ROH ≥ 3 MB (*n* = 7 across all individuals) to accurately estimate load in large ROH for NWR. Dashed lines show the proportions of heterozygous sites with deleterious alleles and homozygous sites with deleterious alleles in non‐autozygous regions of the genome.

Because long ROH from recent inbreeding may harbor high levels of deleterious variants (Szpiech et al., 2013), we tested for enrichment of putatively deleterious alleles in ROH. Compared to shorter ROH, longer ROH had proportionally more homozygous deleterious variants in SWR (linear regression, *p* = 6.25 × 10^−13^ for the ratio of moderate‐impact conserved to low‐impact and *p* = 2.65 × 10^−9^ for the proportion conserved) and NWR (*p* = 3.38 × 10^−5^ for the ratio of moderate‐impact conserved to low‐impact and *p* = 0.017 for the proportion conserved; Figure 3). ROH >1 MB had proportionally more homozygous deleterious variants compared to homozygous variants in non‐autozygous regions of the genome (paired *t*‐test, *p* = 5.60 × 10–5 for homozygous conserved and *p* = 0.018 for homozygous moderate‐impact conserved mutations). By contrast, heterozygous variants in non‐autozygous regions had higher proportions of deleterious alleles compared to homozygous sites in ROH (paired *t*‐test, *p* = 0.028 for homozygous conserved and *p* = 1.53 × 10–9 for homozygous moderate‐impact conserved mutations). Together, these results imply that recent inbreeding may be responsible for a disproportionate share of homozygous genetic load, as heterozygous sites in non‐autozygous regions of the genome are converted to homozygous states in ROH through inbreeding.

To assess the possible functional impacts of genetic load in these populations, we tested for functional enrichment among genes with deleterious variants. Among genes that had missense mutations at evolutionarily conserved sites (RS > 4), NWR had 42 enriched GO terms (Table S3). The most significantly enriched of these included heart development, cellular component morphogenesis, inner ear development, direction of mechanical stimulus involved in sensory perception of sound, epidermal cell differentiation, and cilium organization (all FDR‐corrected *p* < 0.01). Interestingly, three of the top six terms were also enriched among genes with private, missense variants in SWR (heart development, cellular component morphogenesis, and cilium organization). In SWR, the top five most significant GO terms (cilium or flagellum‐dependent cell motility, cilium‐dependent cell motility, heart development, microtubule‐based movement, and biological adhesion; all FDR‐corrected *p* < 0.02) were also significantly enriched among NWR (Table S4).

### 3.3 Simulations of northern white rhino recovery

**FIGURE 4:**
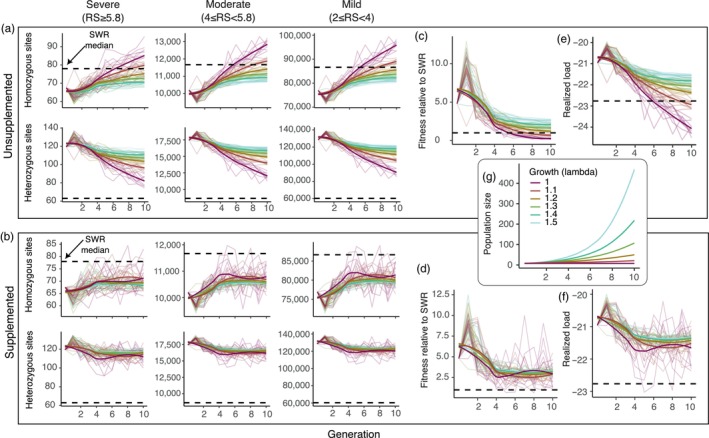
Genetic load and fitness simulated over 10 generations of growth in northern white rhino populations restored from cryopreserved cells. Simulated populations were either founded once in generation 0 (unsupplemented) or founded with subsequent reintroductions of one randomly selected cryopreserved genome each generation (supplemented). Simulation replicates (thin lines; 10 of the 50 replicates are shown) and medians across replicates (thick lines) are colored by growth rate. Dashed black horizontal lines show the median values for SWR genomes. (a, b) Median number of homozygous (upper plots) and heterozygous (lower plots) alleles per genome with severe, moderate, and mild deleterious effects in unsupplemented (a) and supplemented (b) populations. (c, d) Median per‐individual fitness effects of deleterious genetic load each generation relative to the median fitness of SWR genomes. (e, f) Median realized load each generation. (g) Number of individuals per generation in populations with different growth rates.

To estimate the potential fitness impacts of genetic load on a re‐established NWR population, we simulated genetic load and its fitness cost. We started with eight cryopreserved NWR cell lines and simulated 10 generations with and without supplementation of founder genomes in subsequent generations (supplemented and unsupplemented scenarios, respectively). In all scenarios, there was an initial increase in heterozygosity and decrease in homozygosity in generation one, likely resulting from substructure within the group of NWR founders (Sánchez‐Barreiro et al., 2021). Starting in generation two, homozygous load and realized load increased across all deleterious mutation types and fitness decreased, changing most rapidly under the low‐growth scenarios (Figure 4; Figure S10).

In unsupplemented populations with little to no population growth (0–10% per generation), homozygous load and realized load typically exceeded that observed in the SWR population by generations 7–10 (Figure 4a). Under the higher‐growth scenarios (40–50%), the increase in homozygous load typically leveled off around generation five and did not exceed that of SWR over the 10 simulated generations. The number of heterozygous deleterious sites decreased over time as they were largely converted to homozygous states. There was little change over time in the overall number of deleterious alleles per genome, as predicted from population genetic theory (Pinto et al., 2023). This is because, of all polymorphic sites that became fixed by generation 10, a substantial portion (~10–30%) were fixed for the deleterious allele, especially in the low‐growth scenarios (Figure S11). Fitness declined in all unsupplemented populations, but most rapidly in the 0 and 10% growth scenarios, on average falling below the mean value for SWR by generations six to eight (Figure 4c). Fitness declines in the 40–50% growth scenarios typically leveled off before generation 10 and remained higher than the median fitness of SWR until the end of the simulations. The results were qualitatively similar using different selection coefficients (Figure S7).

In populations supplemented with founder genomes in subsequent generations, trends in fitness and genetic load over time were less dependent on population growth (Figure 4b,d; Figure S12). Numbers of homozygous and heterozygous sites were similar for populations with different growth rates (Figure 4b), and there was little separation of the fitness trajectories under different growth rates (Figure 4d). Increases in homozygous load and decreases in both heterozygous load and fitness tended to level off around generation four in all scenarios. Populations with 0% growth showed high stochasticity, likely because the introduction of a random founder genome each generation had a proportionally larger impact on the composition of the very small population. Fitness never consistently fell below the median for SWR, even in the 0–10% growth scenarios (Figure 4d).

## 4 DISCUSSION

### 4.1 Genomic variation reflects both demographic history and contemporary bottlenecks

Demographic history sheds light on the evolutionary context that has shaped the genome‐wide diversity of the two populations, contributing to their current and future viability. Previous inferences of the demographic histories of NWR and SWR showed some disagreement, potentially stemming from unmodeled complexities captured differently by different analyses (Beichman et al., 2017). Phylogenetic and coalescence‐based analysis of single genomes by Moodley et al. (2020) suggested divergence between 580 and 440 KYA with periods of secondary contact until 100–220 KYA, whereas models fit to the folded (i.e., non‐polarized) 2dSFS by Tunstall et al. (2018) suggested divergence between 10 and 20 KYA. In this study, the demographic model that best fit the polarized 2dSFS suggested divergence in isolation ~46–150 KYA (depending on assumptions of generation time) with steady population declines until 296–944 years ago, when both populations began to decline more rapidly. Our divergence time estimate of 46–150 KYA agrees with the end of the period of secondary contact inferred by Moodley et al. (2020). Population declines in both subspecies have also been inferred with microsatellites, with contractions dating from the mid‐Holocene to late Pleistocene (Moodley et al., 2018). Around 6000 years ago the climate reached the Holocene Maximum, marking the end of the African Humid Period and leading to the retreat of the grassland savannah habitat of white rhinos (Drake et al., 2011; Gasse, 2000; Jolly et al., 1998; Skinner & Poulsen, 2016).

The larger historical effective population size of NWR (harmonic mean *N*_e_ = 4759 from T1 to T2) compared to SWR (harmonic mean *N*_*e*_ = 2892) is reflected in higher heterozygosity of NWR genomes and is consistent with the finding of more segregating deleterious alleles and higher masked load in NWR (Bertorelle et al., 2022; Hedrick & García‐Dorado, 2016). The larger number of deleterious alleles in the NWR population are segregating at low frequencies and are largely masked in heterozygosity with, presumably, relatively little impact on fitness. By contrast, the SWR population has more fixed deleterious alleles, both by count and in proportion to the total number of fixed derived alleles. The large number of fixed alleles may reflect the impact of recent bottlenecks. Extreme bottlenecks may lead to the rapid fixation of many alleles across the genome (Dussex et al., 2023), including the conversion of masked to realized load (Bertorelle et al., 2022). The proportional overrepresentation of deleterious alleles among those fixed reflects longer‐term demography. Enrichment of deleterious alleles suggests the impact of genetic drift from small *N*_e_ over the long term (Wilder et al., 2023), as strong genetic drift overwhelms purifying selection, leading to the accumulation and fixation of mildly and moderately deleterious alleles (Lynch et al., 1995).

Estimates of more recent *N*_e_ from the 2dSFS (*N*_*e*_ = 474 and 140 for NWR and SWR, respectively) are likely influenced by the severe population bottlenecks in the past century. The longer duration of the bottleneck by the time the SWR individuals analyzed here were born (1968–2012) resulted in higher inbreeding coefficients, lower heterozygosity, more fixed deleterious alleles, and higher homozygous load compared to NWR, which was still declining when the banked individuals were born (1952–1975). The long tracts of ROH in the SWR genome reflect higher inbreeding in SWR over the past two centuries. ROH tracts stemming from this period may have an outsized impact on fitness, since long ROH are enriched for homozygous deleterious variants relative to shorter ROH and non‐autozygous regions of the genome, which have been exposed to selection in homozygosity for longer periods. Because heterozygous sites in non‐autozygous regions have proportionally more deleterious alleles, future inbreeding in a restored NWR population is likely to bring together regions enriched for heterozygous deleterious variants into homozygosity in long ROH.

For both subspecies, some of the most significantly enriched functions of genes with deleterious mutations were related to cilium‐ or flagellum‐dependent cell motility, which may play a role in sperm motility and fertility. High rates of abnormal sperm have been found in several rhinoceros species (O'Brien & Roth, 2000; Roth et al., 2005; Stoops et al., 2010), including white rhinos (Hermes et al., 2005; Reid et al., 2009), and 81% of males in zoo settings do not sire offspring (Hermes et al., 2005). Abnormal sperm and low sperm motility are hallmarks of inbreeding depression across taxa (Fitzpatrick & Evans, 2009; Gage et al., 2006; Gomendio et al., 2000; Roldan et al., 1998; Wildt et al., 1983; Zajitschek et al., 2009). While it is possible that deleterious mutations in genes important for sperm viability may play a role in inbreeding depression, GO terms directly related to flagellated sperm motility nested within the broader cilium‐ or flagellum‐dependent cell motility term were not significantly enriched, making it difficult to draw direct links between deleterious variation and this trait. The genomic mechanism underlying this commonly observed phenotype of highly inbred populations warrants further investigation.

### 4.2 Viability of a restored NWR population

Despite the comparatively high burden of homozygous deleterious alleles, the SWR population has mounted a remarkable recovery from dozens of individuals, suggesting viability of SWR was not severely limited by genetic load. Recovery of the NWR population will depend on the mitigation of threats, successful implementation of cellular conservation technologies, and on the standing genetic variation of cell lines from 12 NWR individuals. In a population established by few founders, some level of inbreeding will likely be unavoidable, leading to the conversion of masked heterozygous load to realized homozygous load and loss of fitness (Hedrick & García‐Dorado, 2016). Assuming deleterious variants have similar impacts in SWR and NWR genomes, our findings suggest that a NWR population founded by cryopreserved cells from eight unrelated individuals would not be constrained by low fitness if founder genomes are repeatedly reintroduced in subsequent generations. By contrast, a population restored in a single founding event would require rapid population growth to maintain fitness similar to that of SWR. Populations unsupplemented by reintroduced founders showed loss of genetic diversity and increased homozygosity of all deleterious mutation types under every simulated scenario. Fitness from genetic load typically fell below the average for SWR, except when growth was ≥30% per generation (equivalent to an increase from eight to >108 individuals over 10 generations). Given that the SWR population grew from <200 individuals in the early 1900s to 20,000 today (a per‐generation growth rate of ~33–45%), the goal may not be unrealistic. In captive settings, however, SWR populations have seen negative growth rates due to poor reproductive success of captive‐born females (Swaisgood et al., 2006). Low female fertility is thought to be caused by exposure to high phytoestrogen levels during development through the maternal diet and microbial metabolites (Tubbs et al., 2016; Williams et al., 2019). Diet modification can boost fertility and possibly improve population growth (Tubbs et al., 2017), but this issue, along with the potential for low sperm viability, highlights potential barriers to achieving the rapid growth needed to avoid high realized load. Restoration of populations from cryopreserved cell lines allows lost genetic variation to be repeatedly reintroduced as it is lost each generation. This allows for fixed and homozygous deleterious alleles to again be masked in heterozygosity to relieve inbreeding depression, escaping the requirement for rapid growth of the restored population to avoid severe inbreeding depression.

One possible alternative to the reintroduction of founders may be to use a genetic rescue strategy, hybridizing NWR and SWR to introduce genetic variation into a restored NWR population. At least one hybrid is known, though it never reproduced (Saragusty et al., 2016). However, the subspecies have been isolated far longer than standard guidelines for avoiding outbreeding depression (Frankham et al., 2019), and loci that may cause genetic incompatibilities are difficult to predict from genomic data, making reintroduction of founders a potentially safer strategy.

Forward genomic simulations are increasingly used in conservation for understanding the constraints on population viability and recovery, enabling exploration of population dynamics and outcomes under a given set of assumptions. For example, genomic simulations have been used to assess the potential recovery of endangered vaquitas (Robinson et al., 2022), estimate purging in Channel Island foxes (Robinson et al., 2018), model genetic rescue through facilitated gene flow (Kyriazis et al., 2021), reveal adaptation in response to fisheries (Therkildsen et al., 2019), and predict the impact of habitat loss on genomic erosion (Pinto et al., 2023). Simulations have even been proposed as a potential source of data for training models to predict IUCN extinction risk categories and recovery potential of species (van Oosterhout et al., 2022a, 2022b). Here, we used forward‐simulated populations from empirically derived genetic variation (Speak et al., 2023), a novel approach that is especially relevant when the founders of a future restored population are known.

Despite the advancement of genomic simulation tools and our ability to estimate the impact of genome‐wide genetic variation in non‐model species, there are many caveats to the interpretation of both. Quantifying the effect of mutations on individual fitness is notoriously complex and plagued by a general lack of data (Grueber & Sunnucks, 2022). To estimate fitness variation from genomic variants, we applied selection and dominance coefficients estimated from human genomic data and evolutionary constraints (Henn et al., 2016; Peischl et al., 2018). We used a simple evolutionary model and assumed that mutations have similar fitness effects in NWR and SWR. However, the fitness effects of mutations can differ between species, populations, or even individuals (Grossen et al., 2020; Mee & Yeaman, 2019; Zhang et al., 2020), and evolutionary constraint can reflect past selection on loci that no longer affect fitness (Huber et al., 2020). Given the moderate depth of coverage of the genome, heterozygous sites are likely underestimated (Kishikawa et al., 2019), which may impact the predicted fitness trajectory of the more heterozygous NWR genomes in particular. Beneficial mutations, which were not modeled in this study, also likely influence individual variation in fitness. Populations managed under human care and with ART may experience different selective pressures, some of which may lead to maladaptation (van Oosterhout et al., 2022a, 2022b; Williams & Hoffman, 2009). For these reasons, estimates of the fitness effects of genetic variants are imprecise, and management decisions based solely on presumably functional genetic variation of individuals can be misleading (Kardos et al., 2021; Ralls et al., 2020). As genomic data representing diverse taxa continue to grow, and especially when coupled with databases linking survival and lifetime reproduction (Grueber & Sunnucks, 2022), so too will our power to link genotype to genomic health, fitness, and population viability and eventually use genomics‐informed strategies to manage realized load (Speak et al., 2023). These insights will be key for applying conservation strategies, including rapidly advancing in vitro conservation technologies, with greater precision.

Our simulations highlight the value of biobanks for restoring populations from even a small number of individuals, and demonstrate how cellular‐based conservation strategies can be powerful resources for rescuing populations from the brink of extinction. However, these technologies are not panaceas, and on their own will not solve the extinction crisis and global loss of intraspecific genetic variation. Cellular‐based restoration and de‐extinction may cost many times more than traditional conservation strategies that protect natural populations, and those protections will still be needed after restoration. Rather, these established conservation methods can be augmented by new cellular‐based tools (Phelan et al., 2021). Technological advances may remove some hurdles, but at present, application of the technologies envisioned in this study required a surrogate species for gestation and become feasible because viable cell lines from multiple individuals are available. Biobanking of viable somatic and germline cells today represents a significant opportunity for conserving biological diversity, building the capacity to help restore genetic variation to critically small or declining populations in the future.

## CONFLICT OF INTEREST STATEMENT

The authors have no conflicts of interest to disclose.

## Supporting information


Appendix S1:

## Data Availability

Raw sequence data for this study are available at NCBI: PRJNA394025; ENA: ERS6578958, ERS6578959, ERS6578960, ERS6578961, ERS6578962, ERS6578963, ERS6578964, ERS6578965, ERS6578966. Scripts are available on github at: https://github.com/apwilder/WhiteRhino_GeneticLoad.
