# Supplementary material for: Genetic load and viability of a future restored northern white rhino population
Source: Evol Appl. 2024 Apr 11;17(4):e13683. doi: 10.1111/eva.13683 (PMC11009427; doi:10.1111/eva.13683)
Supplement: Supplementary file 1 — Appendix S1: [file EVA-17-e13683-s001.docx]

**Supplemental Tables and Figures**

Genetic load and viability of a future restored northern white rhino population

# Tables

| Table S1. Sample and sequencing details for modern NWR and SWR individuals. Reads from samples in batch 2 were randomly subsampled to a mean depth of 14.32x. * NWR individual without cell lines. | | | | | | | | |  |
| --- | --- | --- | --- | --- | --- | --- | --- | --- | --- |
| Sample | Population | Birth year | Birth type | Sex | Batch | Reads | Mean depth | SD. depth | |
| NWR_KB3731 | NWR | 1952 | Wild | F | 1 | 2.44E+08 | 15.18 | 39.09 | |
| NWR_KB6571 | NWR | 1952 | Wild | M | 1 | 2.21E+08 | 13.75 | 29.68 | |
| NWR_KB8174 | NWR | 1965 | Wild | F | 1 | 2.50E+08 | 15.54 | 36.77 | |
| NWR_KB5763* | NWR | 1972 | Wild | F | 1 | 2.49E+08 | 15.47 | 29.50 | |
| NWR_KB5764 | NWR | 1972 | Wild | F | 1 | 2.39E+08 | 15.10 | 45.65 | |
| NWR_KB9947 | NWR | 1972 | Wild | M | 1 | 2.37E+08 | 14.83 | 37.89 | |
| NWR_KB5766 | NWR | 1973 | Wild | M | 1 | 2.49E+08 | 15.52 | 41.17 | |
| NWR_KB8175 | NWR | 1974 | Wild | F | 1 | 1.75E+08 | 11.01 | 30.58 | |
| NWR_KB9939 | NWR | 1975 | Wild | M | 1 | 2.10E+08 | 12.94 | 20.73 | |
| SWR_KB6974 | SWR | 1958 | Wild | F | 1 | 2.26E+08 | 13.85 | 25.26 | |
| SWR_KB13306 | SWR | 1961 | Wild | M | 1 | 2.19E+08 | 13.52 | 26.14 | |
| SWR_KB5892 | SWR | 1963 | Wild | F | 1 | 2.39E+08 | 15.06 | 41.27 | |
| SWR_KB7062 | SWR | 1963 | Wild | F | 1 | 2.33E+08 | 14.34 | 29.77 | |
| SWR_109 | SWR | 1968 | Wild | F | 2 | 3.69E+08 | 23.38 | 49.67 | |
| SWR_110 | SWR | 1969 | Wild | F | 2 | 3.55E+08 | 22.21 | 40.24 | |
| SWR_104 | SWR | 2008 | Captive | F | 2 | 3.61E+08 | 22.55 | 35.40 | |
| SWR_102 | SWR | 2009 | Captive | F | 2 | 3.05E+08 | 19.30 | 57.40 | |
| SWR_103 | SWR | 2010 | Captive | F | 2 | 3.10E+08 | 19.35 | 37.15 | |
| SWR_105 | SWR | 2011 | Captive | F | 2 | 3.18E+08 | 20.22 | 61.82 | |
| SWR_106 | SWR | 2011 | Captive | F | 2 | 3.84E+08 | 24.29 | 44.01 | |
| SWR_107 | SWR | 2011 | Captive | F | 2 | 3.96E+08 | 25.07 | 49.52 | |
| SWR_108 | SWR | 2012 | Wild | F | 2 | 2.86E+08 | 18.46 | 63.34 | |

| Table S2. Parameter estimates for the best fitting model estimated from CLAIC in GADMA. The model includes an ancestral population that splits into two populations at time T1, with two subsequent periods (following T2 and T3) of size change in both populations. Parameter estimates give point estimates and 95% lower and upper confidence intervals (LCI and UCI, respectively) estimated from block bootstrapped data. Time parameters are scaled to generations and years assuming 8 years per generation (Tunstall et a. 2018) and 25.5 years per generation (Moodley et al. 2020). | | | | | | | | | | |
| --- | --- | --- | --- | --- | --- | --- | --- | --- | --- | --- |
|  |  | Generations ago | | | Years ago (8 yrs/gen.) | | | Years ago (25.5 yrs/gen.) | | |
| Parameter | Definition | Point estimate | LCI | UCI | Point estimate | LCI | UCI | Point estimate | LCI | UCI |
| Nanc | Ancestral population size | 14,024 | 12,833 | 15,180 | – | – | – | – | – | – |
| T1 | Time since split | 5,864 | 5,326 | 6,355 | 46,912 | 42,608 | 50,840 | 149,532 | 135,813 | 162,053 |
| NWR1 | Size of NWR at T1 | 13,456 | 12,306 | 14,579 | – | – | – | – | – | – |
| SWR1 | Size of SWR at T1 | 4,945 | 4,523 | 5,356 | – | – | – | – | – | – |
| NWR1.1 | Size of NWR just before T2 | 1,042 | 954 | 1,128 | – | – | – | – | – | – |
| SWR1.1 | Size of SWR just before T2 | 1,533 | 1,402 | 1,659 | – | – | – | – | – | – |
| m12_1 | Proportion of migrants in NWR from SWR between T1 and T2 | 0 | 0 | 0 | – | – | – | – | – | – |
| m21_1 | Proportion of migrants in SWR from NWR between T1 and T2 | 0 | 0 | 0 | – | – | – | – | – | – |
| T2 | Time of first size change | 37 | 33 | 41 | 296 | 264 | 328 | 944 | 842 | 1,046 |
| NWR2 | Size of NWR at T2 | 192 | 176 | 208 | – | – | – | – | – | – |
| SWR2 | Size of SWR at T2 | 349 | 319 | 378 | – | – | – | – | – | – |
| m12_2 | Proportion of migrants in NWR from SWR between T2 and T3 | 0 | 0 | 0 | – | – | – | – | – | – |
| m21_2 | Proportion of migrants in SWR from NWR between T2 and T3 | 0 | 0 | 0 | – | – | – | – | – | – |
| T3 | Time of second size change | 28 | 25 | 31 | 224 | 200 | 248 | 714 | 638 | 791 |
| NWR3 | Size of NWR at T2 | 474 | 433 | 513 | – | – | – | – | – | – |
| SWR3 | Size of SWR at T2 | 140 | 128 | 152 | – | – | – | – | – | – |
| m12_3 | Proportion of migrants in NWR from SWR after T3 | 0.03094 | 0.03089 | 0.03094 | – | – | – | – | – | – |
| m21_3 | Proportion of migrants in SWR from NWR after T3 | 0 | 0 | 0 | – | – | – | – | – | – |

| Table S3. GO terms significantly over-represented among genes with moderate-impact mutations at evolutionarily conserved (RS>4) that were private in NWR. | | | | |
| --- | --- | --- | --- | --- |
| GO ID | Description | Genes with term | Corrected p-value | Also enriched in SWR? |
| GO:0007507 | heart development | 560 | 5.08E-03 | * |
| GO:0032989 | cellular component morphogenesis | 830 | 4.25E-03 | * |
| GO:0048839 | inner ear development | 191 | 7.08E-03 |  |
| GO:0050910 | detection of mechanical stimulus involved in sensory perception of sound | 15 | 7.17E-03 |  |
| GO:0009913 | epidermal cell differentiation | 194 | 6.06E-03 |  |
| GO:0044782 | cilium organization | 337 | 5.62E-03 | * |
| GO:0000904 | cell morphogenesis involved in differentiation | 570 | 5.24E-03 |  |
| GO:0042472 | inner ear morphogenesis | 101 | 5.21E-03 |  |
| GO:0048667 | cell morphogenesis involved in neuron differentiation | 456 | 5.47E-03 |  |
| GO:0007156 | homophilic cell adhesion via plasma membrane adhesion molecules | 119 | 6.60E-03 | * |
| GO:0022610 | biological adhesion | 765 | 6.23E-03 | * |
| GO:0000902 | cell morphogenesis | 724 | 5.90E-03 |  |
| GO:0008544 | epidermis development | 282 | 5.83E-03 |  |
| GO:0043583 | ear development | 212 | 6.14E-03 |  |
| GO:0007605 | sensory perception of sound | 145 | 5.99E-03 |  |
| GO:0007155 | cell adhesion | 760 | 5.69E-03 | * |
| GO:0030216 | keratinocyte differentiation | 140 | 7.93E-03 |  |
| GO:0060271 | cilium assembly | 318 | 8.47E-03 |  |
| GO:0050954 | sensory perception of mechanical stimulus | 175 | 8.72E-03 |  |
| GO:0007017 | microtubule-based process | 627 | 8.78E-03 | * |
| GO:0030855 | epithelial cell differentiation | 547 | 9.26E-03 |  |
| GO:0001539 | cilium or flagellum-dependent cell motility | 29 | 9.99E-03 | * |
| GO:0060285 | cilium-dependent cell motility | 29 | 9.99E-03 | * |
| GO:0048562 | embryonic organ morphogenesis | 299 | 0.014 |  |
| GO:0001895 | retina homeostasis | 62 | 0.014 |  |
| GO:0007420 | brain development | 786 | 0.013 |  |
| GO:0007368 | determination of left/right symmetry | 131 | 0.013 |  |
| GO:0031175 | neuron projection development | 692 | 0.013 |  |
| GO:0060322 | head development | 833 | 0.014 |  |
| GO:0042471 | ear morphogenesis | 116 | 0.014 |  |
| GO:0048666 | neuron development | 835 | 0.015 |  |
| GO:0050982 | detection of mechanical stimulus | 50 | 0.015 |  |
| GO:0007018 | microtubule-based movement | 248 | 0.015 | * |
| GO:0070886 | positive regulation of calcineurin-NFAT signaling cascade | 16 | 0.018 |  |
| GO:0106058 | positive regulation of calcineurin-mediated signaling | 16 | 0.018 |  |
| GO:0003007 | heart morphogenesis | 260 | 0.018 |  |
| GO:0120035 | regulation of plasma membrane bounded cell projection organization | 706 | 0.020 |  |
| GO:0048568 | embryonic organ development | 437 | 0.019 |  |
| GO:0007423 | sensory organ development | 561 | 0.020 |  |
| GO:0098742 | cell-cell adhesion via plasma-membrane adhesion molecules | 199 | 0.021 |  |
| GO:0009855 | determination of bilateral symmetry | 138 | 0.021 |  |
| GO:0072359 | circulatory system development | 871 | 0.020 |  |
| GO:0009799 | specification of symmetry | 139 | 0.022 |  |
| GO:0031344 | regulation of cell projection organization | 713 | 0.023 |  |
| GO:0070268 | cornification | 76 | 0.026 |  |
| GO:0045494 | photoreceptor cell maintenance | 41 | 0.027 | * |
| GO:0048598 | embryonic morphogenesis | 591 | 0.027 |  |
| GO:0031032 | actomyosin structure organization | 117 | 0.029 |  |
| GO:0090596 | sensory organ morphogenesis | 270 | 0.028 |  |
| GO:0007409 | axonogenesis | 378 | 0.029 |  |
| GO:0055002 | striated muscle cell development | 143 | 0.029 |  |
| GO:0043588 | skin development | 253 | 0.031 |  |
| GO:0048747 | muscle fiber development | 56 | 0.031 |  |
| GO:0060119 | inner ear receptor cell development | 49 | 0.030 |  |
| GO:0055001 | muscle cell development | 153 | 0.031 |  |
| GO:0048812 | neuron projection morphogenesis | 493 | 0.031 |  |
| GO:0031581 | hemidesmosome assembly | 14 | 0.039 |  |
| GO:0120039 | plasma membrane bounded cell projection morphogenesis | 498 | 0.039 |  |
| GO:0098609 | cell-cell adhesion | 396 | 0.039 |  |
| GO:0007610 | behavior | 583 | 0.040 |  |
| GO:0048858 | cell projection morphogenesis | 502 | 0.045 |  |
| GO:0051961 | negative regulation of nervous system development | 329 | 0.046 |  |

| Table S4. GO terms significantly over-represented among genes with moderate-impact mutations at evolutionarily conserved (RS>4) that were private in SWR. | | | | |
| --- | --- | --- | --- | --- |
| GO ID | Description | Genes with term | Corrected p-value | Also enriched in NWR? |
| GO:0001539 | cilium or flagellum-dependent cell motility | 29 | 6.12E-04 | * |
| GO:0060285 | cilium-dependent cell motility | 29 | 6.12E-04 | * |
| GO:0007507 | heart development | 560 | 0.012 | * |
| GO:0007018 | microtubule-based movement | 248 | 0.010 | * |
| GO:0022610 | biological adhesion | 765 | 0.014 | * |
| GO:0007099 | centriole replication | 19 | 0.015 |  |
| GO:0007155 | cell adhesion | 760 | 0.013 | * |
| GO:0060122 | inner ear receptor cell stereocilium organization | 37 | 0.013 |  |
| GO:0007017 | microtubule-based process | 627 | 0.017 | * |
| GO:0007156 | homophilic cell adhesion via plasma membrane adhesion molecules | 119 | 0.017 | * |
| GO:0051056 | regulation of small GTPase mediated signal transduction | 314 | 0.022 |  |
| GO:0044782 | cilium organization | 337 | 0.022 | * |
| GO:0045494 | photoreceptor cell maintenance | 41 | 0.022 | * |
| GO:0051298 | centrosome duplication | 28 | 0.021 |  |
| GO:0060088 | auditory receptor cell stereocilium organization | 18 | 0.035 |  |
| GO:0098534 | centriole assembly | 24 | 0.036 |  |
| GO:0032989 | cellular component morphogenesis | 830 | 0.037 | * |

# Figures


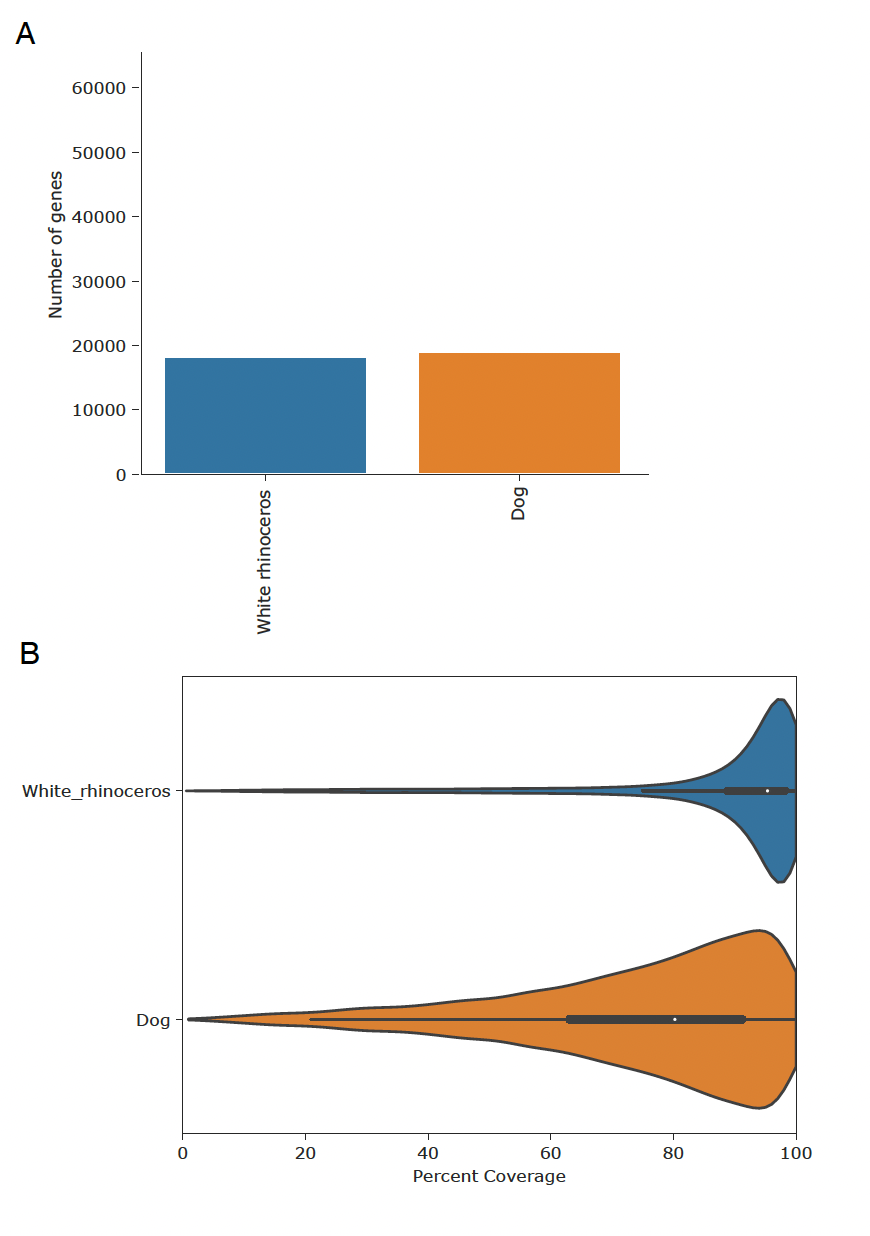


Figure S1. (A) The number of protein-coding genes identified in the white rhino genome annotation was similar to that of the dog genome annotation. (B) Coverage of protein coding genes was higher in the rhino annotation compared to the dog.


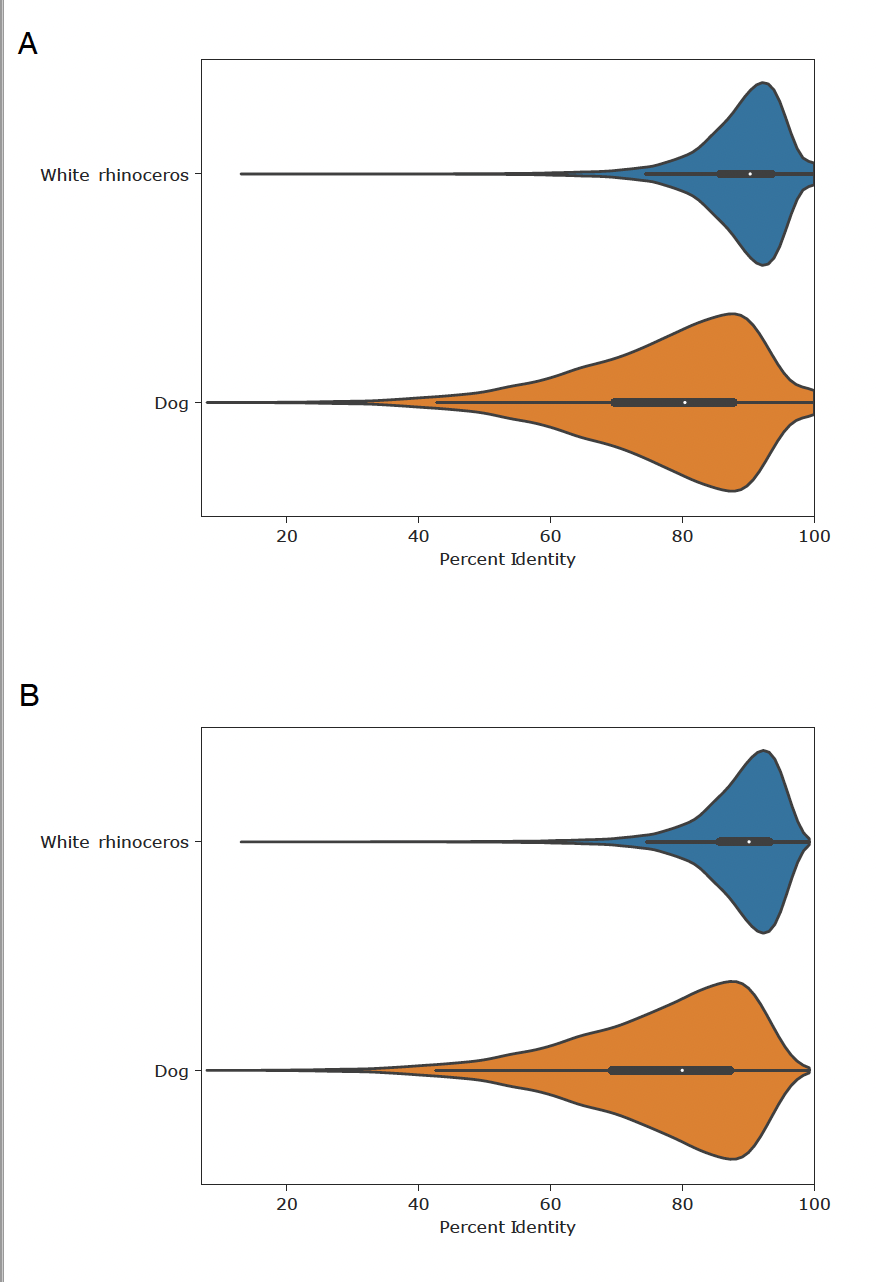


Figure S2. Percent Identity of overall annotation (A) and protein-coding genes only (B) of white rhino compared to dog annotation.


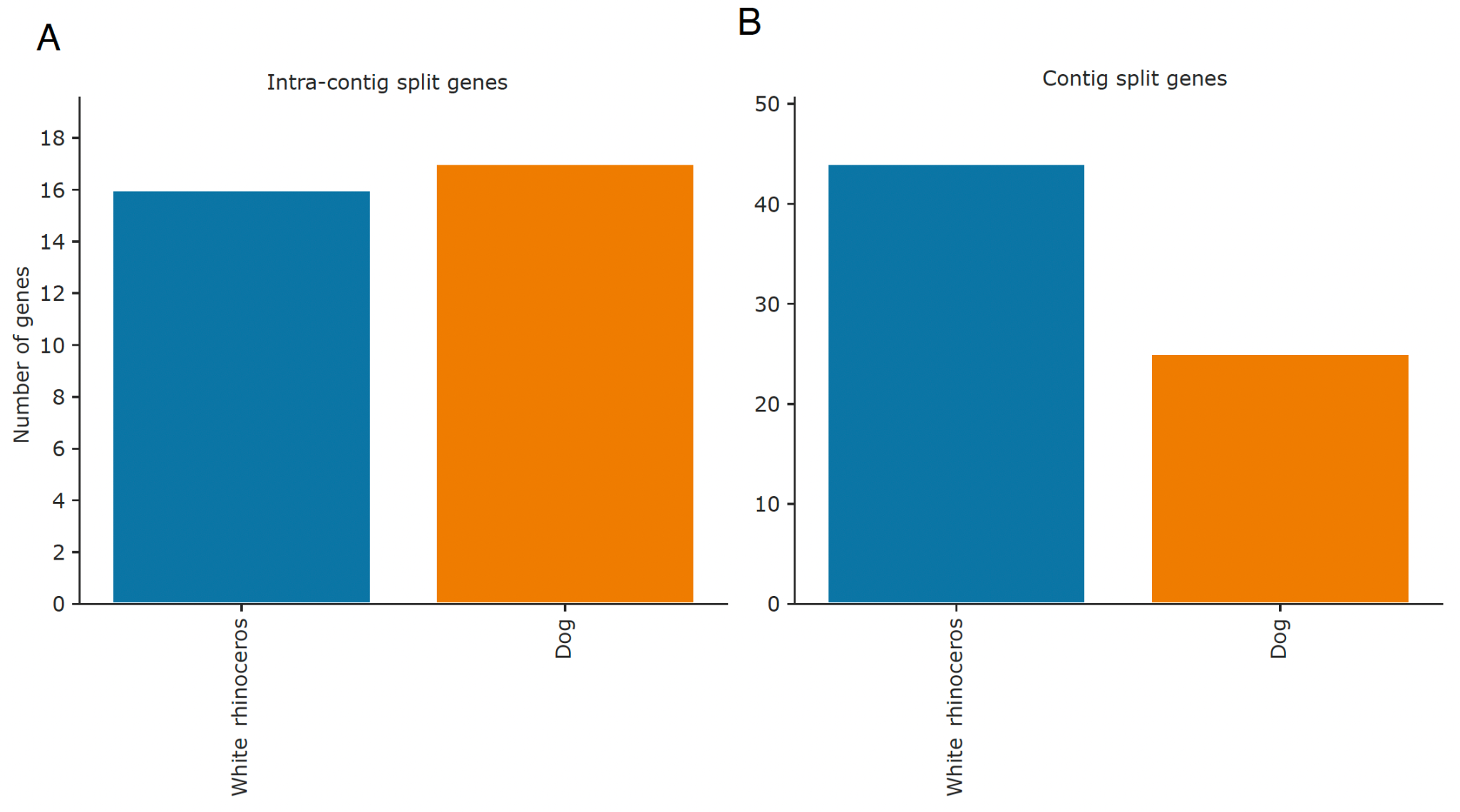


Figure S3. Number of genes split within contigs (A) and across contigs (B) in white rhino annotation compared to dog.


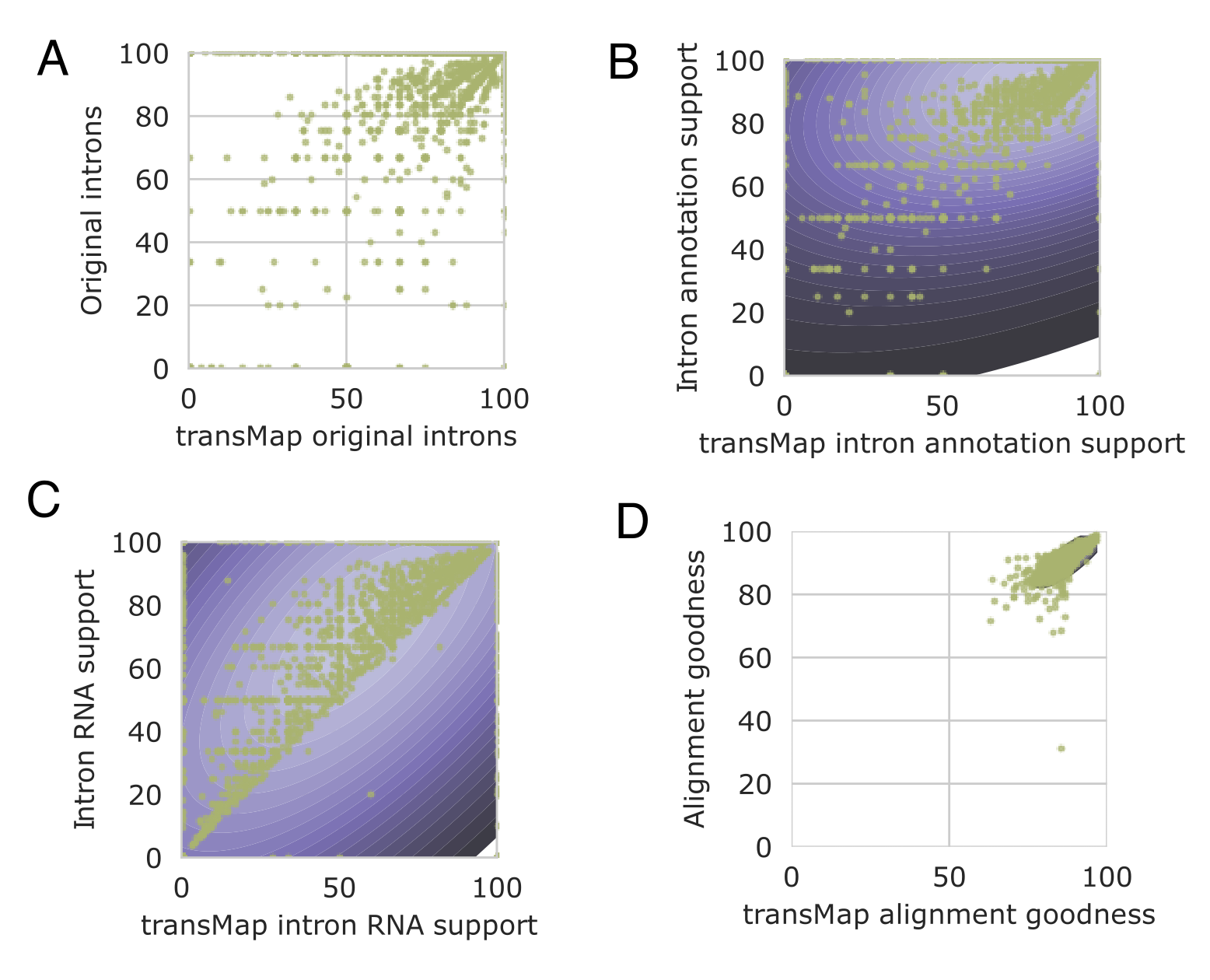


Figure S4. AUGUSTUS metric improvements for 3,051 transcripts in the white rhinoceros genome annotation. 14,167 transMap transcripts were chosen.


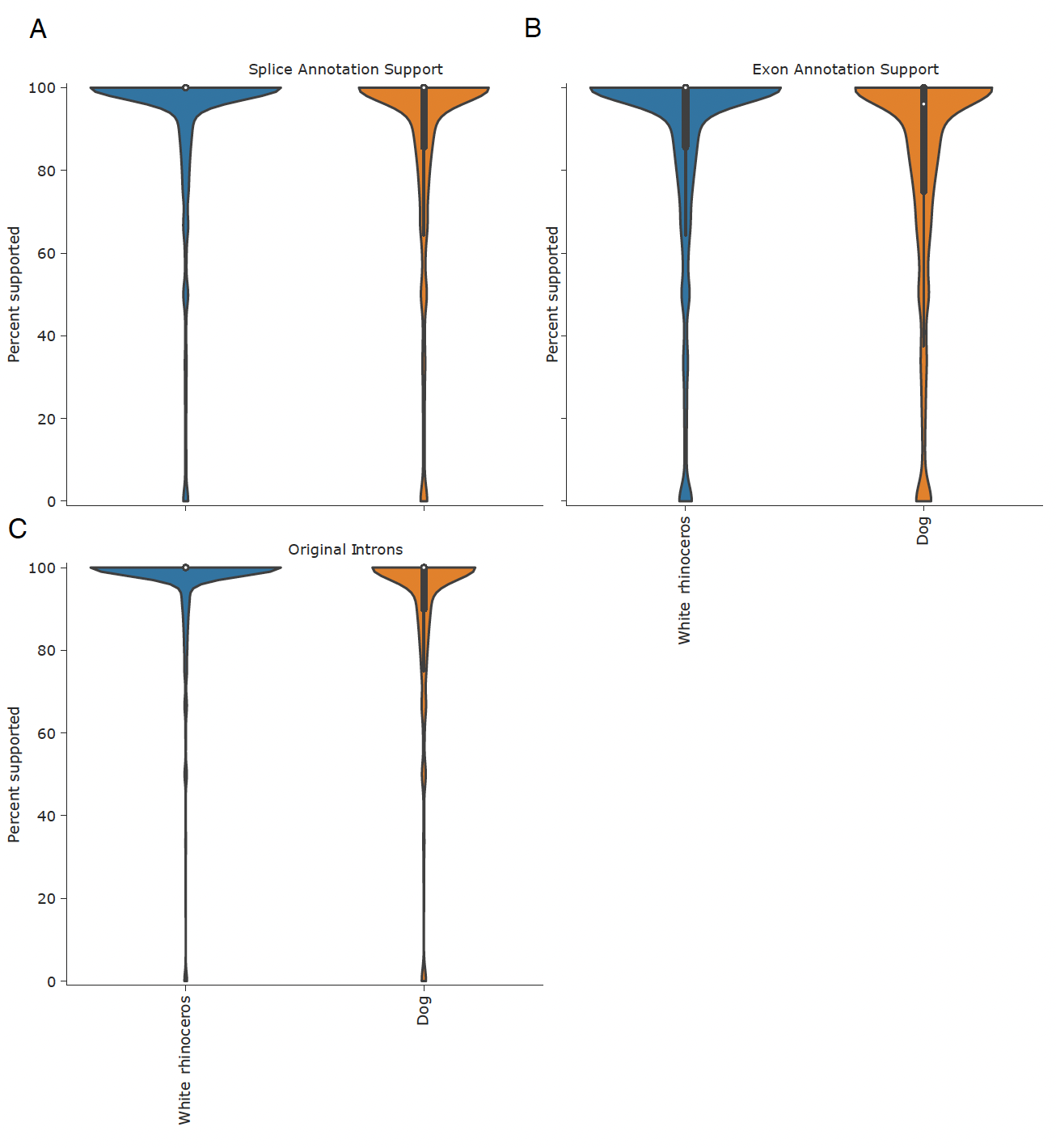


Figure S5. Reference annotation support for splice annotation, exon annotation and introns of protein coding genes in the white rhino genome annotation compared to dog.


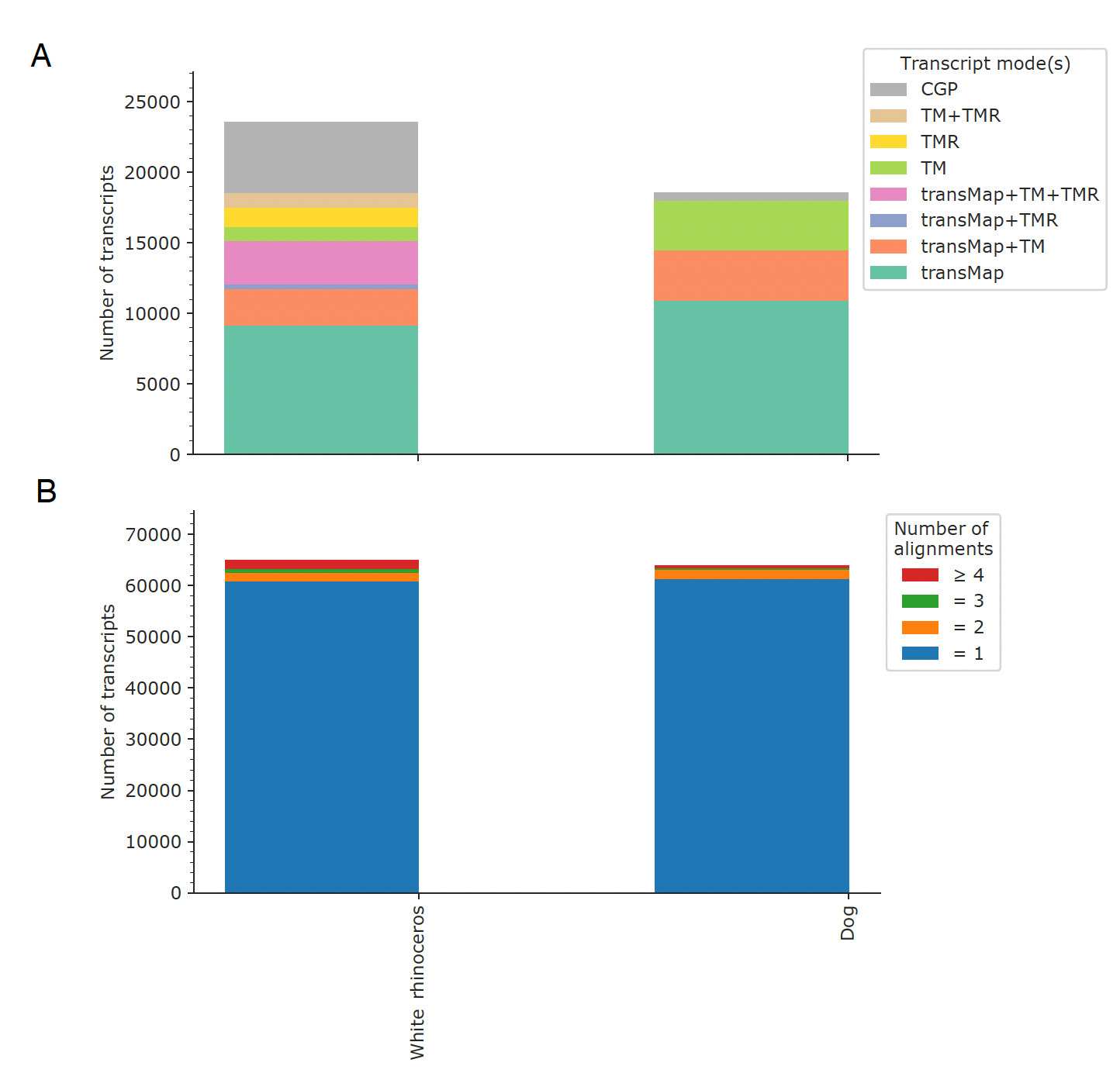


Figure S6. (A) Transcript modes in protein coding consensus gene set and (B) proportion of transcripts that have multiple alignments for the white rhino annotation compared to dog.


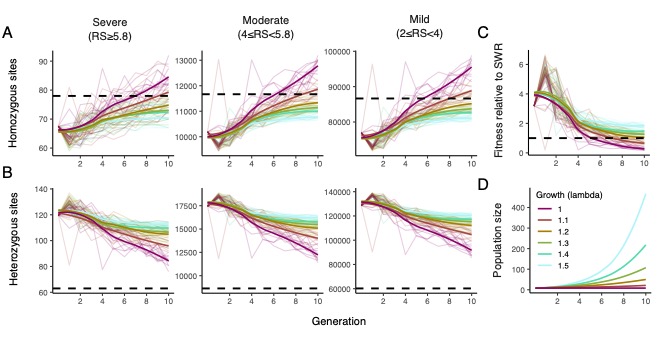


Figure S7. Genetic load and fitness trends of northern white rhino populations restored from cryopreserved cells were similar using different selection coefficients *s*_neutral_ = 0, *s*_mild_ = −0.024, *s*_moderate_ = −0.032, and *s*_severe_ = −0.072. Per-generation population growth rates ranged from lambda=1.0 to 1.5 (0-50%), and populations were not supplemented with additional founder genomes in later generations. Simulation replicates (thin lines) and medians across replicates (thick lines) are colored by growth rate. Dashed black horizontal lines show the median values for SWR genomes. (A and B) Median number of homozygous (A) and heterozygous (B) alleles per genome with severe, moderate and mild deleterious effects. (C) Median per-individual fitness effects of deleterious genetic load each generation relative to the median fitness of SWR genomes. (D) Number of individuals per generation in populations with different growth rates


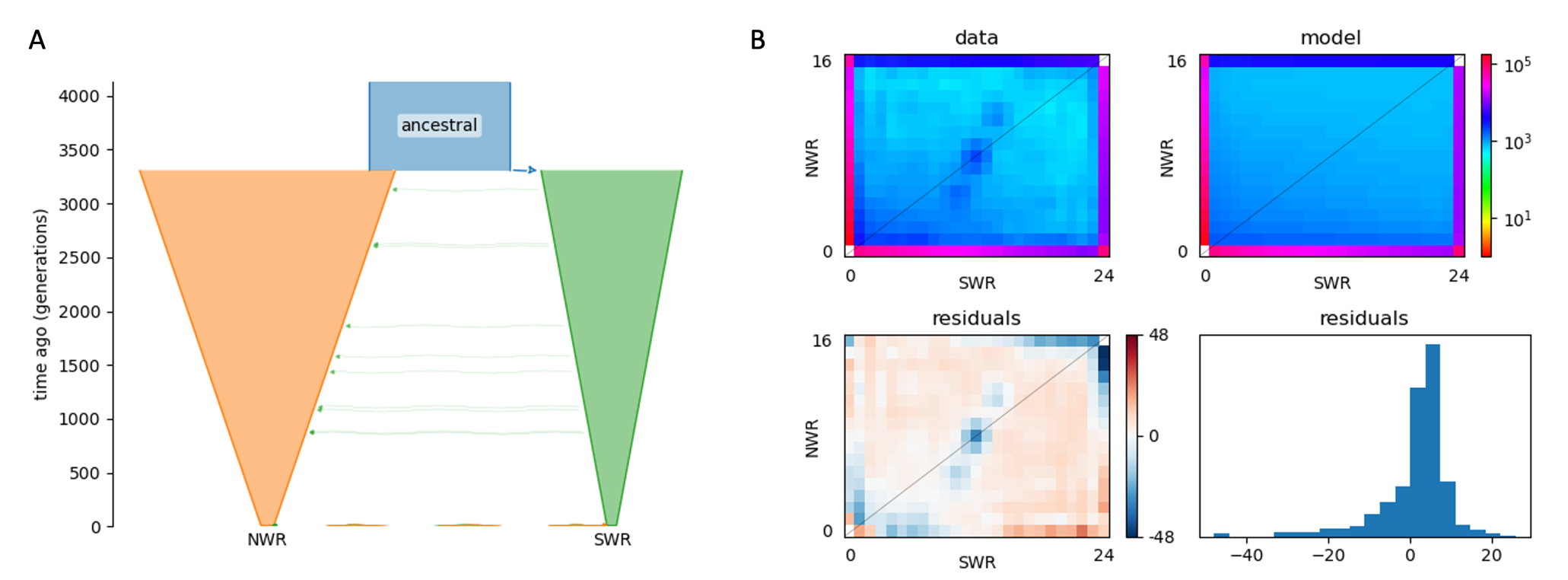


Figure S8. A) Diagram depicting the best fitting demographic model selected using CLAIC in GADMA. B) Observed (data) and simulated (model) 2dSFS and the residuals between model versus data and their distribution.


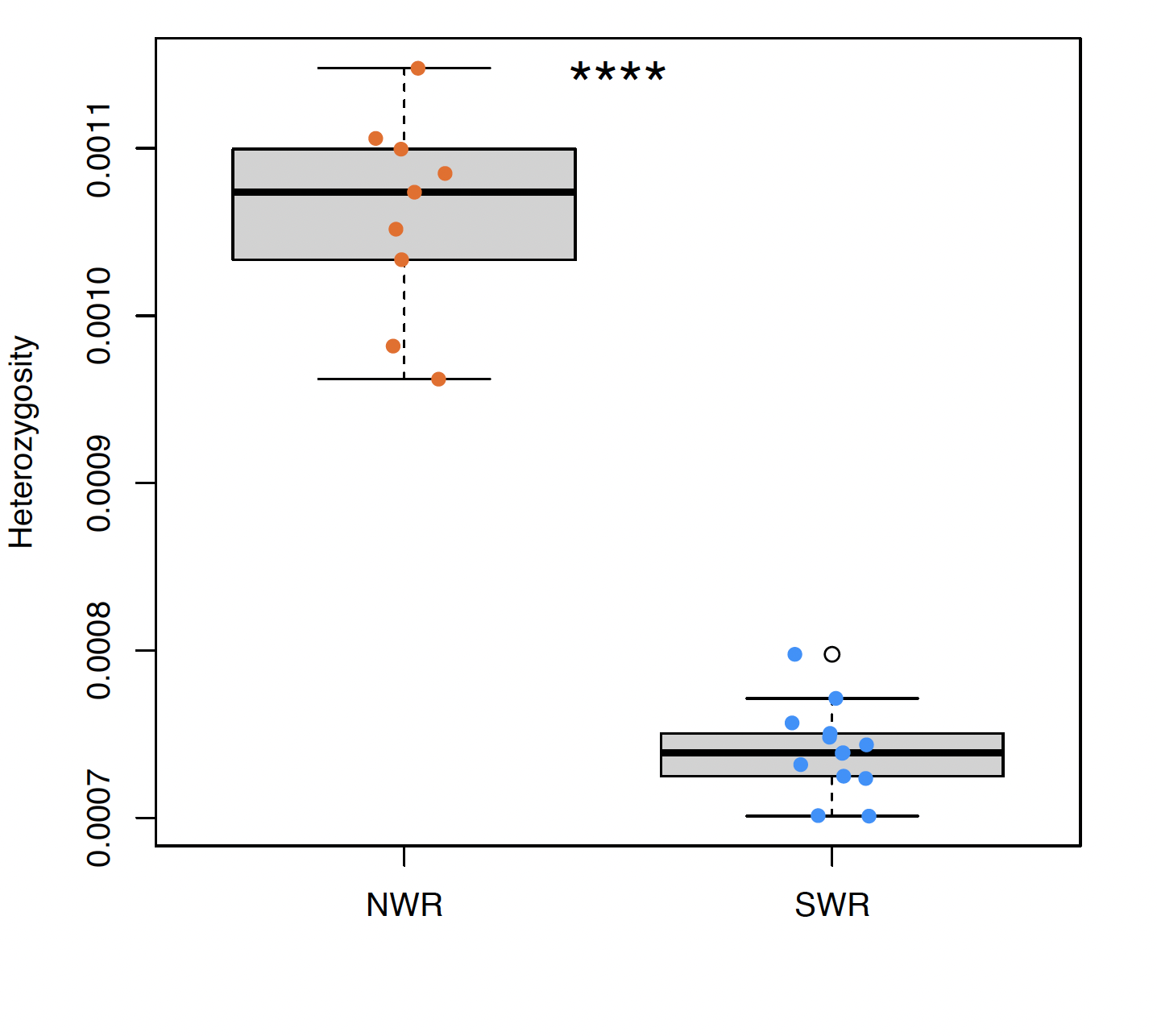


Figure S9. Heterozygosity of northern white rhino genomes (mean=1.06×10-3; standard error=2.00×10-5) was significantly higher than southern white rhino genomes (mean=7.41×10-4; standard error=7.32×10-6; p=2.27e-13) as represented by asterisks.


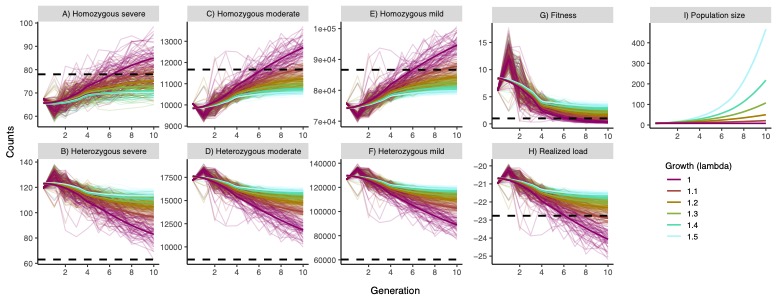


Figure S10. Simulations of genetic load and fitness trends of northern white rhino populations restored from cryopreserved cells across 50 replicates. Per-generation population growth rates ranged from lambda=1.0 to 1.5 (0-50%), and populations were not supplemented with additional founder genomes in later generations. Simulation replicates (thin lines) and medians across replicates (thick lines) are colored by growth rate. Dashed black horizontal lines show the median values for SWR genomes. (A–F) Median number of homozygous (A, C and E) and heterozygous (B, D and F) alleles per genome with severe, moderate and mild deleterious effects. (G) Median per-individual fitness effects of deleterious genetic load each generation relative to the median fitness of SWR genomes. (H) Median per-individual realized load each generation relative to the median of SWR genomes. (I) Number of individuals per generation in populations with different growth rates


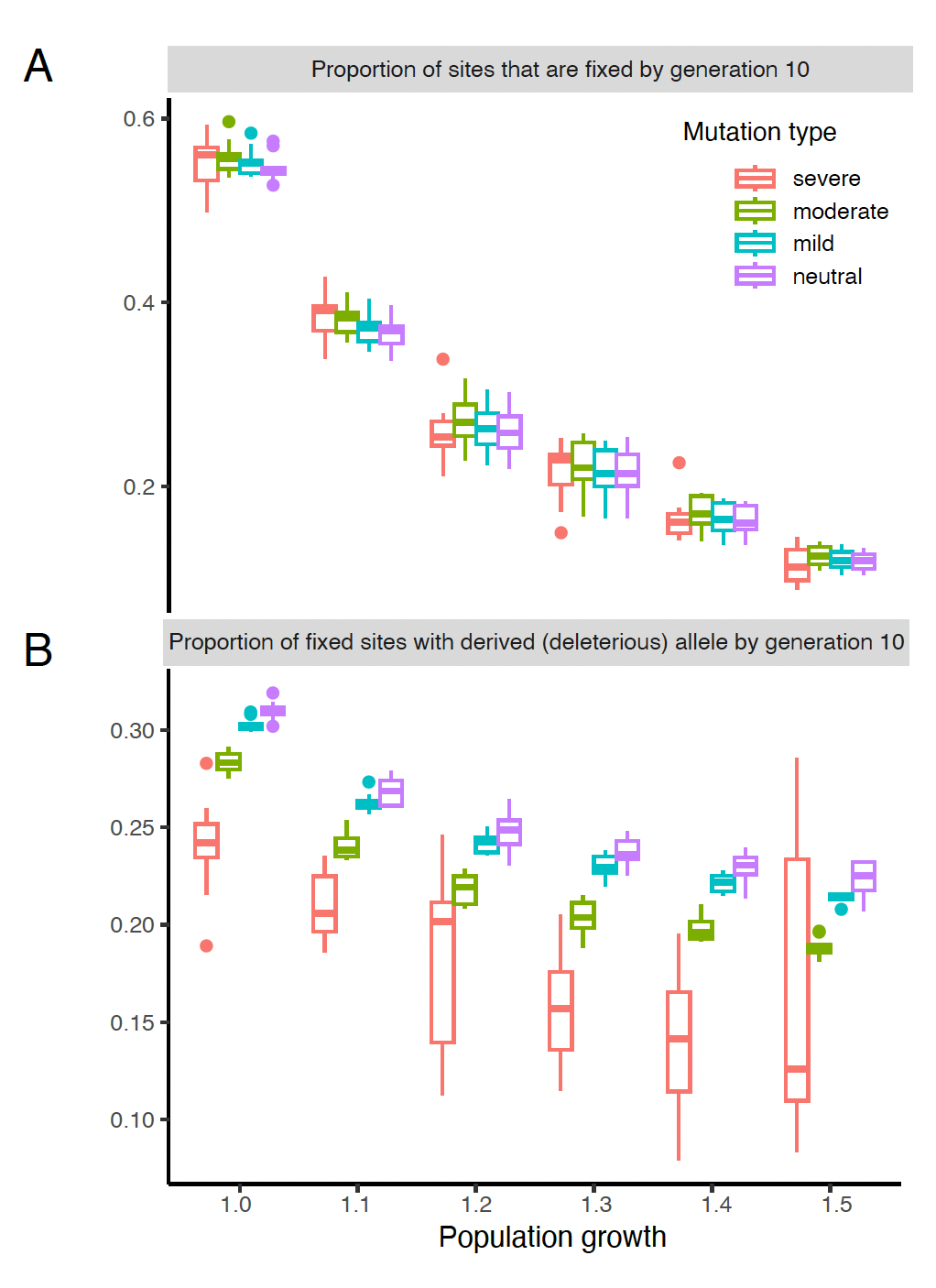


Figure S11. Fixation and loss of deleterious alleles under different population growth simulations in a recovering northern white rhino population without supplementation of founders in later generations. (A) The proportion of alleles that are fixed by generation 10 of simulations. More sites are fixed under low population growth scenarios. (B) The proportion fixed for the derived allele at fixed sites where the derived allele has a deleterious impact that is severe, moderate, mild or neutral on fitness. 10-30% of fixed alleles are deleterious, with more deleterious alleles fixed under low growth scenarios.


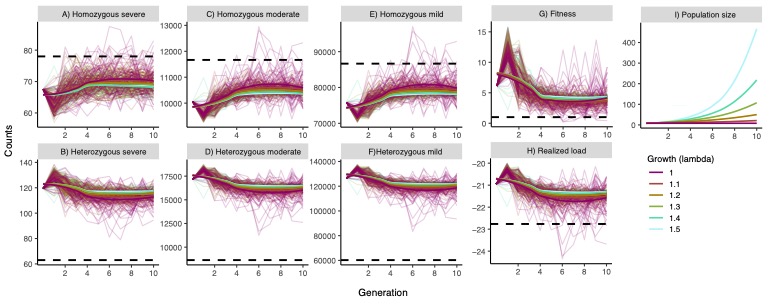


Figure S12. Simulations of genetic load and fitness trends of northern white rhino populations restored from cryopreserved cells across 50 replicates. Per-generation population growth rates ranged from lambda=1.0 to 1.5 (0-50%), and populations were supplemented with additional founder genomes in later generations. Simulation replicates (thin lines) and medians across replicates (thick lines) are colored by growth rate. Dashed black horizontal lines show the median values for SWR genomes. (A–F) Median number of homozygous (A, C and E) and heterozygous (B, D and F) alleles per genome with severe, moderate and mild deleterious effects. (G) Median per-individual fitness effects of deleterious genetic load each generation relative to the median fitness of SWR genomes. (H) Median per-individual realized load each generation relative to the median of SWR genomes. (I) Number of individuals per generation in populations with different growth rates.
